# Structure and Computational Studies of New Sulfonamide Compound: {(4-nitrophenyl)sulfonyl}tryptophan

**DOI:** 10.3390/molecules27217400

**Published:** 2022-10-31

**Authors:** Florence Uchenna Eze, Chigozie Julius Ezeorah, Blessing Chinweotito Ogboo, Obinna Chibueze Okpareke, Lydia Rhyman, Ponnadurai Ramasami, Sunday Nwankwo Okafor, Groutso Tania, Simeon Atiga, Thomas Ugochukwu Ejiyi, Mirabel Chinasa Ugwu, Chiamaka Peace Uzoewulu, Jude Ikechukwu Ayogu, Ogechi Chinelo Ekoh, David Izuchukwu Ugwu

**Affiliations:** 1 Department of Pure and Industrial Chemistry, University of Nigeria, Nsukka 410001, Nigeria; 2 Department of Chemistry and Biochemistry, University of South Carolina, Columbia, SC 29208, USA; 3 Department of Chemistry, State University of New York at Buffalo, Buffalo, NY 14260, USA; 4 School of Science, University of Waikato, Private Bag 3105, Hamilton 3240, New Zealand; 5 Computational Chemistry Group, Department of Chemistry, Faculty of Science, University of Mauritius, Réduit 808037, Mauritius; 6 Centre for Natural Product Research, Department of Chemical Sciences, University of Johannesburg, Doornfontein, Johannesburg 2028, South Africa; 7 Department of Pharmaceutical and Medicinal Chemistry, University of Nigeria, Nsukka 410011, Nigeria; 8 School of Chemical Sciences, University of Auckland, Private Bag 92019, Auckland 1142, New Zealand; 9 Department of Chemistry, North Carolina State University, Raleigh, NC 27607, USA; 10 Department of Chemistry, Evangel University, Akaeze 491104, Nigeria

**Keywords:** L-tryptophan, sulfonamide, crystal, DFT, docking

## Abstract

Synthesis of sulfonamide through an indirect method that avoids contamination of the product with no need for purification has been carried out using the indirect process. Here, we report the synthesis of a novel sulfonamide compound, ({4-nitrophenyl}sulfonyl)tryptophan (DNSPA) from 4-nitrobenzenesulphonylchloride and L-tryptophan precursors. The slow evaporation method was used to form single crystals of the named compound from methanolic solution. The compound was characterized by X-ray crystallographic analysis and spectroscopic methods (NMR, IR, mass spectrometry, and UV-vis). The sulfonamide N-H NMR signal at 8.07–8.09 ppm and S-N stretching vibration at 931 cm^−1^ indicate the formation of the target compound. The compound crystallized in the monoclinic crystal system and P2_1_ space group with four molecules of the compound in the asymmetric unit. Molecular aggregation in the crystal structure revealed a 12-molecule aggregate synthon sustained by O-H⋯O hydrogen bonds and stabilised by N-H⋯O intermolecular contacts. Experimental studies were complemented by DFT calculations at the B3LYP/6-311++G(d,p) level of theory. The computed structural and spectroscopic data are in good agreement with those obtained experimentally. The energies of interactions between the units making up the molecule were calculated. Molecular docking studies showed that DNSPA has a binding energy of −6.37 kcal/mol for *E. coli* DNA gyrase (5MMN) and −6.35 kcal/mol for COVID-19 main protease (6LU7).

## 1. Introduction

**Figure 1:**
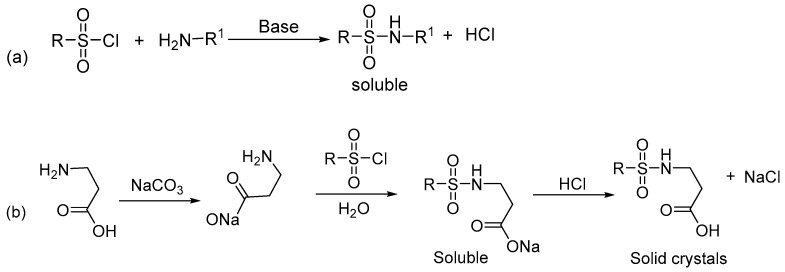
Synthesis of sulfonamide using (**a**) direct and (**b**) indirect approaches.

Sulfonamides constitute a useful group of compounds with extensive applications in medicinal and synthetic chemistry [1,2,3,4], with the sulfonamide functional group being a major building block for many therapeutic molecules [3,4] and natural products. Consequently, these applications have paved the way for the continuous search and design of new drug formulations based on this class of compounds. There are several methods for synthesizing sulfonamides with attendant hurdles, some of which could be surmounted [5,6,7,8]. Sulfonamides generation using organic alcohols or esters, halides, aryl boronic acids and aminosulfonation with the aid of catalysts have been encountered [9,10,11,12]. Recently, there has been a report on the use of transition metal fluoride salts on solid molecular sieve support for synthesizing sulfonamide compounds [13]. Heterogenous catalysts afford the synthetic procedure a greener approach—bypassing the tedious aqueous work-up step [13,14]. Challenges usually encountered in most of these synthetic methods involving transition metal-based catalysts lie in the possible contamination of the desired product by inseparable catalysts, thereby hindering the maximum use of the product in the pharmaceutical industry. Efforts have been made to generate pure sulfonamides using other green and sustainable methods [15,16,17,18,19,20,21] by utilizing eco-friendly solvents such as water or ethanol coupled with complete solvent recovery [15,16,17]. Known procedure utilizing the direct reaction of sulfonyl chloride and amino compounds in the presence of a base exists (Figure 1a) [22]. Owing to the precursors’ high reactivity and the simplicity of the reaction, this approach has attracted much attention [21,22,23,24,25]. The challenge of using excess base to scavenge the acid formed is a major drawback facing this method as the product is soluble in the acid. This has prompted the indirect approach using amino carboxylic acid species, which affords soluble sodium carboxylate intermediate that gives insoluble pure products upon reaction with sulfonyl chloride and hydrochloric acid (Figure 1b) [26,27,28]. No further purification is required in this approach as the product comes out very pure, unlike other procedures highlighted above. Given the stated reasons, we approached our synthesis using carboxylic amine and sulfonyl chloride. 

In addition to the experimental studies on sulfonamides, theoretical methods have also been used for research works on sulfonamide chemistry. Recently, Tehari et al. [29] used B3LYP/6-31+G(d,p) method to study the Li^+^ and Na^+^ ion affinities with hydroxymethylated 1,4-disubstituted-1,2,3-triazole-based sulfonamides. Bonyad and co-workers [30] investigated the structure and spectroscopic parameters of 1-amide 4-sulfonamide-1,2,3-triazole derivatives using the B3LYP/6-31G(d) method. The same group [31] extended their studies by exploring the structural and spectroscopic aspects of 1-ester 4-sulfonamide-1,2,3-triazole scaffolds based on the B3LYP/6-31G(d) method. In 2020, Verma et al. [32] carried out a critical review on the antibacterial activities of sulfonamide containing heterocyclic derivatives and relevant structure-activity relationships (SAR) studies. Hassan and co-workers [33] reported on 3d multifunctional metal chelates of sulfonamide and their studies included computations with the WB97XD/LanLD2Z method for structure optimization. In view of these and a careful review of the literature, we have complemented the current research using the B3LYP/6-311++G(d,p) method to have additional insights at molecular level.

Moreover, Computer-Aided Drug Design (CADD) over the years, has brought significant breakthroughs in the field of drug discovery [34,35,36]. Its critical roles in the cost-effective identification of promising drug candidates, reduction of the use of animal models in pharmacological research, facilitating the rational design of novel and safe drug candidates, and in drug repurposing have made it an attractive design approach [37]. In the conventional process of drug discovery, it has been estimated that it takes average of 10–15 years [38,39] and 1.8 billion USD [40] to bring a new drug into the market. CADD has drastically reduced both the time and cost thereby speeding up the process of drug discovery. Many studies based on in silico tools have virtually screened small molecule databases and published a huge amount of information on new drug discoveries for the coronavirus disease (COVID-19) [41]. Various studies showed that the indole-derivatives have shown promising anti-SARS CoV-2 activity [41]. Specifically, Borgio and his co-workers have determined through molecular docking studies that an indole-derivative has a docking score of −9.84 kcal/mol involving a hydrogen bond formation with Gly79 of SARS CoV-2 helicase. Thus, the compound interfered with the viral replication potential of helicase, making it an efficient inhibitor of SARS CoV-2 helicase [42]. Not many in silico studies have been done on sulfonamides against COVID-19. However, Liu et al. [42] have clearly demonstrated through in silico studies, that the isatin derivative containing sulfonamide group improved the inhibitory activity against SARS CoV-2. The sulfonamide compound extended hydrogen bonds with residues Gly143 and Cys145 of SARS Coronavirus Main Proteinase (3CLpro), with a docking score of 8.70 using Glide 5.5 software. The docking results also revealed the crucial role of N-1 and C O at position 2 of isatin nucleus in hydrogen bond formation. Similarly, delavirdine, an anti-HIV drug containing sulfonamide group, has been repurposed as anti-SARS CoV-2 through in silico studies [43]. It exhibited a marked inhibitory activity against modelled SARS CoV-2 RdRp structure forming hydrogen bonds with Ala576 and Asn 582, with a docking score of −8.5. Due to insufficient in silico studies of sulfonamides against SARS CoV-2 and the established linkage that they are potential SARS CoV-2 inhibitors, we aim to evaluate the anti- SARS CoV-2 activity of our synthesized sulfonamide.

This work is a continuation of an ongoing project in our group where we have worked on several carboxamide derivatives bearing sulfonamide functionalities. Their in vitro antimalarial [44], antitrypanosomal [45,46], anti-inflammatory and analgesic [47], COX-II inhibitor [48], and anthelmintic [49] properties have been examined. In each case, they have shown great activities against the studied target. However, our group have not isolated their single crystals for studies; hence, we started a novel synthesis of sulfonamide species and their single-crystal studies to look at their bonding patterns and molecular interactions. In this paper, we are reporting the synthesis of ({4-nitrophenyl}sulfonyl)tryptophan (DNSPA) from L-tryptophan and 4-nitrobenzenesulfonylchloride using the indirect method described above and its investigation by physical and theoretical techniques. Computational studies were employed to supplement experimental data.

## 2. Results

### 2.1. X-ray Crystallographic Structure Analysis

**Figure 2:**
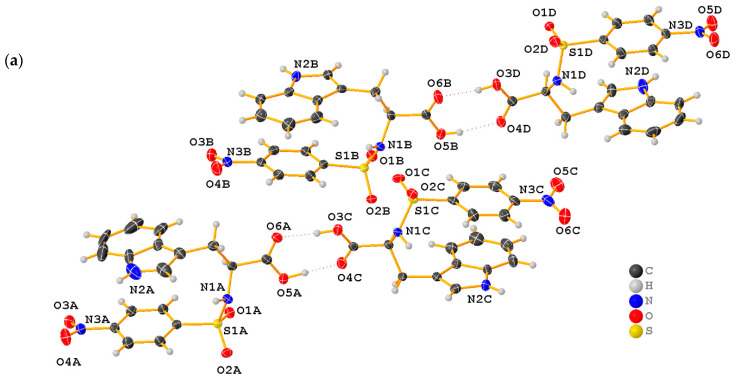

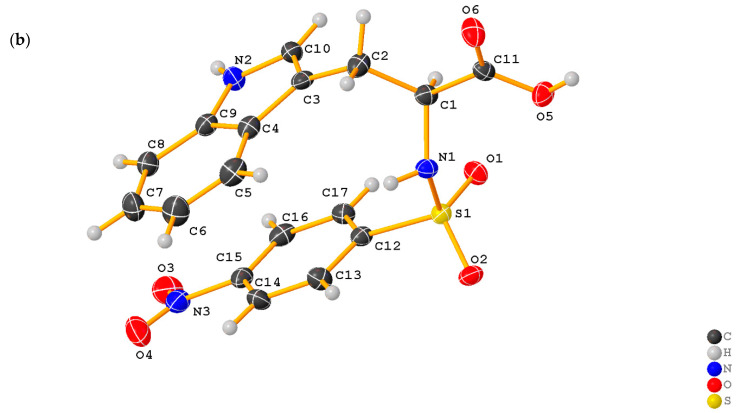
(**a**) Crystal structure of ((4-nitrophenyl)sulfonyl)tryptophan (DNSPA) showing four independent molecules in the asymmetric unit connected by C-H∙∙∙O intermolecular hydrogen bond. (**b**) Molecular structure of one molecule of DNSPA. Ellipsoids are drawn at 50% probability.

The crystal structure of DNSPA presented in Figure 2a shows that the compound crystallized with four similar but independent molecules in the asymmetric unit. The molecules have been designated A-D, with each pair of the molecules connected by carboxyl-C-H⋯O(carboxyl) intermolecular hydrogen bonds. The molecular structure in Figure 1b shows a central SO_2_ moiety connected to tryptophan and p-nitrophenyl substituents. The geometry around the S1 atom is a distorted tetrahedral with angles subtended at the S1 atom ranging from a narrow N1-S1-O2 (105.23(15)), involving a single bonded N1 and a doubly bonded O2 atom, to a wide O1-S1-O2 (120.47(16)) involving two doubly bonded O1, O2 atoms [50]. The orientation of the DNSPA structure is such that the p-nitrophenyl substituent and the indole fragment of the tryptophan substituent are cis to the central SO_2_ moiety and the amino acid end of the tryptophan substituent. The central SO_2_ plane is separated from the plane of the amino acid residue, the indole residue, and p-nitrophenyl substituents by dihedral angles of magnitude 73.62, 63.77, and 62.29°, respectively. The torsion angles between the planes of the sulfonamide O2C-S1C-N1C-C1C, O1C-S1C-N1C-C1C, and C12C-S1C-N1C-C1C, are 167.2(3)°, 37.5(3)° and −78.4(3)°, respectively.

The sulfonamide S1-N1 bond length of 1.624(3) Å, is slightly shorter than the expected average N-S bond length (1.771(4) Å) [51,52], probably due to the influence of the electronegative oxygen atoms on the S1 atom resulting in a decrease in the N1-S1 bond length. The N3-C15 bond length of the p-nitro substituent of 1.473(4) Å is longer than the N2-C10 (1.356(7) Å) and N2-C9 (1.358(8) Å) bonds of the aromatic indole ring. The shortened bond lengths of the indole N2-C10 and N2-C9 are probably due to aromaticity. Selected geometric parameters; experimental: bond lengths, bond angles, and torsion angles; calculated bond lengths and angles for DNSPA are presented in Tables S1–S4, respectively. The root means square deviations (RMSDs) for the bond lengths and angles between the computed and experimental structures are 0.020 Å and 1.5°, respectively. The compound shares some similarities and striking differences with another sulfonamide published by Ullah Khan et al. [53]. Observation of the crystal structure shows that both are isomers of each other, with the ortho-nitro group in the published and the para-nitro group in DNSPA. In the reported crystal, the crystal system and space group are tetragonal and P4_1_2_1_2, while that of DNSPA are monoclinic and P2_1_. The interaction with the unknown solvent observed by the authors was not present in the present structure as it crystallized without interaction with any solvent in the asymmetric unit.

**Figure 3:**
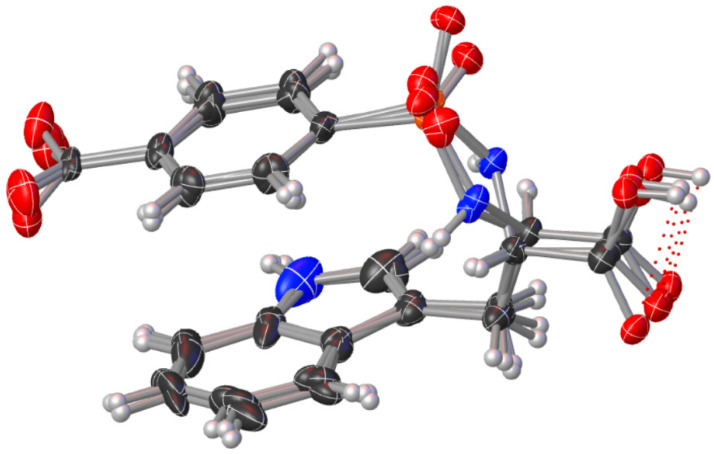
Structure overlay of the four independent DNSPA molecules showing only slight structural differences.

To analyze the structural similarities and differences between the four independent molecules in the crystal lattice of DNSPA, a structure overlay of the four structures A-E, presented in Figure 3 shows that there are no significant structural differences between the independent structures. However, there are differences in the structural orientation of the sulfonamide S1-N1-C1 functionality with the S1-N1-C1 bond angle in structure B being larger by a magnitude of 2° when compared to the corresponding angles in structures A, C and D. Another slight difference in geometric parameters between the four independent molecules was observed in the magnitude of the indole C9-N2 bonds with an average of 1.378 Å in structures B, C and D but recorded as 1.355 Å in structure A. This is probably due to a slight disorder in the heterocyclic indole ring in structure A.

**Figure 4:**
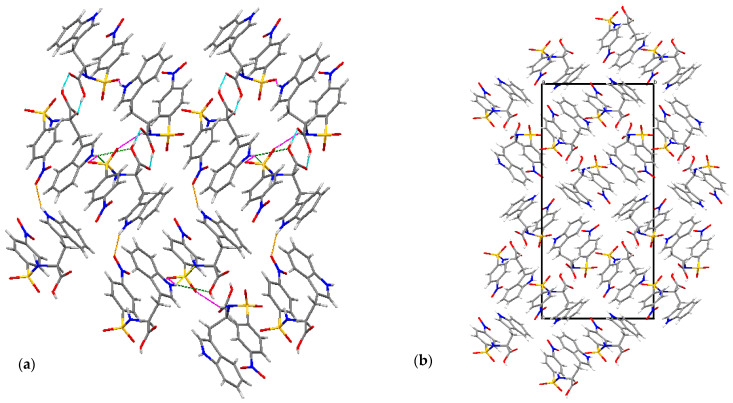
Molecular packing in the crystal structure of DNSPA: (**a**): 12-molecule aggregate supramolecular tape linked by indole-N-H⋯O(sulfonyl) (magenta dotted lines) and indole-N-H⋯O(p-nitrophenyl) (orange lines) and sustained by a bunch of carboxyl-O-H⋯O(carboxyl) (blue), indole-N-H⋯N(Sulfanamide) (yellow) and indole-N-H⋯O(carbonyl) (green) interactions. (**b**) molecular aggregation and crystal packing along the b-axis.

The molecular packing of DNSPA in Figure 4 consists of two molecule aggregates connected by intermolecular indole-N-H⋯O(sulfonyl) hydrogen bonds and linked to two other molecules of the compound by carboxyl-O-H⋯O(carboxyl) intermolecular hydrogen bonds to give a four-molecule aggregate stabilized by an intermolecular hydrogen bond between the carboxylic acid species [50]. The four-molecule aggregate is further linked to two other molecules through the indole-N-H⋯O(p-nitrophenyl) intermolecular interaction to give a 6-molecule aggregate synthon. This six-molecule aggregate synthon is linked to another six-molecule aggregate synthon by indole-N-H⋯O(carboxyl) to give a twelve-molecule 3-dimensional supramolecular synthon. The supramolecular architecture in the structure is stabilized by O6⋯H-O3, O5-H⋯O4, O6⋯H-O3, and O5-H⋯O4 intermolecular contacts with dimensions; 1.776, 1.880, 1.788, and 1.655 Å, respectively. The geometric parameters characterizing the hydrogen bonding interactions in the structure are presented in Table 1.

The X-ray structure was used as input geometry to perform single-point computations in the gas phase on the dimeric structure of DNSPA to calculate the electronic interaction energy of the monomeric structures. The interaction energies are −9.7 kcal/mol [B3LYP/6-311++G(d,p)] and −12.4 kcal/mol [B3LYP-D3(BJ)/6-311++G(d,p)]. The interaction energy for the tetrameric structure in the gas phase, as per Figure 2, was also calculated. The interaction energies among the four monomeric units are +8.4 kcal/mol [B3LYP/6-311++G(d,p)] and −16.2 kcal/mol [B3LYP-D3(BJ)/6-311++G(d,p)]. The negative interaction energy leads to stabilization with respect to the monomeric unit. In addition, based on these values, the unfavourable interaction energy of about +8.6 kcal/mol among the dimers of the stacking units using the B3LYP-D3(BJ)/6-311++G(d,p) method is expected.

### 2.2. Electronic Spectra

**Figure 5:**
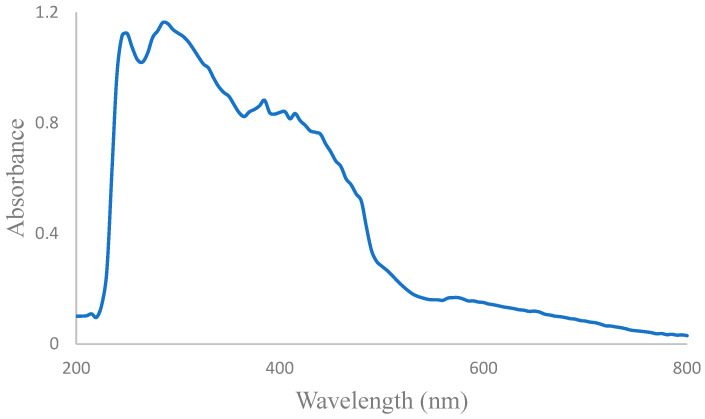
Experimental UV spectra of DNSPA.

The UV-vis spectra (Figure 5) of the synthesized compound showed peaks at 245, 290, 385, 420, and 575 nm. The π-π* transitions were assigned 245 and 290 nm. The n–π* transitions of the sulfonamide were observed at 385, 420, and 575 nm.

The simulated UV spectra of DNSPA is displayed in Figure S1, and three absorption peaks at 186, 217, and 295 nm are observed. The electronic transition at 186 nm, corresponding mainly to H-5 → L + 2 (57%), is attributed to the π-π* of the 4-nitrophenyl moiety. The peak at 217 nm arises due to the π-π* of the indole moiety, and this corresponds to H → L + 7 (36%). Two major transitions are observed at 295 nm corresponding to H-4 → L (50%) and H-3 → L (46%), where the orbitals indicate the n–π* transition of the sulfonamide moiety along with π-π* transition of the 4-nitrophenyl moiety. The electronic transitions’ details and molecular orbitals are collected in Table 2. Notably, there is a slight difference between the experimental and theoretical UV, which can be attributed to free rotations in the solution.

### 2.3. Vibrational Analysis

Figure S2a,b display the experimental and simulated IR spectra. The computed vibrational modes (cf. Table S5) were assigned based on potential energy distribution (PED) analysis using VEDA4 software [54,55]. The sulfonamide S-N stretching vibration mode is observed at 931 cm^−1^. This is in agreement with the computed vibrational mode observed at 847 cm^−1^ and the reported vibrational mode of S-N stretching (935 cm^−1^) [56]. The two vibrational modes of SO_2_ fall within 1125–1330 cm^−1^ [57,58]. The bands observed at 1330 and 1157 cm^−1^ are due to asymmetric and symmetric stretching vibrations of SO_2,_ while the computed S=O stretching is observed at 1298 cm^−1^. Other vibrational modes of SO_2_ also occur, including wagging, scissoring, rocking, and twisting vibrations. These vibrations correspond to the bands observed at 530, 582, 424 and 461 cm^−1^, respectively.

The C-N vibrational modes are observed at 1458 and 1478 cm^−1^. This is corroborated by the computational studies, which show similar C-N stretching vibrations at 1449 and 1479 cm^−1^. The C-H stretching vibrational modes of para-disubstituted benzene occur in the range 3000–3100 cm^−1^ [56]. In our case, it was recorded at 3035 and 3105 cm^−1^. The bending vibrations of the para-disubstituted aromatic C-H bonds are expected in the region 790–840 cm^−1^ [56], but 821 and 846 cm^−1^ bands were observed in the IR spectrum. The in-plane and out-of-plane deformation modes were observed at 1243, 1274, 1288 cm^−1,^ and 932, 959 cm^−1^, respectively. These are in agreement with the expected values (>1000 cm^−1^ and <1000 cm^−1^ for in-plane and out-of-plane deformation modes, respectively) [56]. The stretching vibrations of C-S appear in the region of 800–600 cm^−1^_,_ and this was computed as 742 cm^−1^, while the spectrum reveals 777 and 735 cm^−1^.

The secondary amine N-H band appears as a single weak band in the region of 3350–3310 cm^−1^ [56]. The observed band in the present work for N-H stretching vibration is 3291 cm^−1^. The N-H stretching from the indole moiety is computed at 3670 cm^−1^, while the N-H stretching from the sulfonamide moiety corresponds to 3496 cm^−1^. The N-H bend is observed at 1602 cm^−1^ in IR. The C-O and C=C stretching vibrations are experimentally observed at 1940 and 1579 cm^−1,^ respectively. The theoretical calculations for these vibrations are in agreement with experimental values; the studies showed that C-O stretching vibration is seen at 1810 cm^−1^ while the C=C stretching vibrations correspond to the peak at 1582 cm^−1^.

### 2.4. ^13^C and ^1^H NMR Spectral Analysis

The ^13^C and ^1^H NMR spectra of DNSPA (Figures S4 and S5) were recorded in methanol solvent with tetramethylsilane (TMS) as the internal standard. The experimental and calculated values of ^13^C and ^1^H NMR spectra for correlations are given in Table S6, with the numbering of atoms given in Figure of DNSPA as part of Table S4.

The observed ^13^C NMR spectra revealed values >100 ppm. This agrees with chemical shift values obtained in organic molecules [59], which guarantees proper interpretation. The aliphatic carbon C34 (in the ^13^C-NMR spectrum) is assigned the upfield signal at 27.49 ppm, and this is supported by computation where the chemical shift is predicted at 31.88 ppm. The aromatic signals observed in the range of 116.80 to 125.56 ppm were assigned to the heterocyclic indole aromatic ring. The downfield signals at 125.78 and125.97 ppm are due to the effect of the electronegative nitrogen N9 on C29 and C32, respectively. The carbon chemical shifts are predicted in the region 115.88 to 143.46 ppm, whereby 143.46 ppm is assigned to C29, and 131.58 ppm is assigned to C32. However, the experimental chemical shift of C18 is more upfield (116.80 ppm) and is predicted at 115.88 ppm. The para-substituted nitrobenzene ring carbons have signals appearing in the range of 135.66 to 144.88 ppm. The nitro group’s and para-sulphonyl’s effects led to these higher downfield signals than typical aromatic carbon values. The predicted chemical shifts are observed in the region from 132.19 to 158.81 ppm. The signal at 173.19 ppm is assigned to C26. The far downfield signal of C26 is as expected due to the carboxylic oxygens, and this is in agreement with the predicted chemical shift at 182.26 ppm.

For DNSPA, 15 protons in different environments were identified using the ^1^H NMR spectrum. The aliphatic protons of C34 and C14 are responsible for the upfield signals at 3.20, 3.23, and 3.24 ppm. This is confirmed by the theoretical findings where the protons of C34 are computed at 3.11 and 3.34 ppm, and the proton of C14 is observed at 3.65 ppm. The indole aromatic protons are observed at 6.91–7.45 ppm, and the computed chemical shifts are observed in the range of 6.91 to 7.95 ppm. Appearing at 7.77–8.09 ppm are the signals of the protons of the 4-nitrophenyl moiety, and these agree with the predicted chemical shifts, which are observed in the range of 7.27 to 8.66 ppm. The N-H protons are observed downfield between 7.74 and 7.76 ppm. The computed N-H signal of the indole moiety is at 7.87 ppm, while the N-H proton of the sulfonamide group is predicted at 4.32 ppm. This discrepancy is because the two N-H signals depend on the environment and solvent. As a result, comparing these protons is complex from both experiment and theory [60]. Besides the N-H protons, it can be concluded that the observed chemical shifts of the ^1^H and ^13^C NMR agree with the theoretical results (cf. Table S6).

### 2.5. Molecular Docking Studies

**Figure 6:**
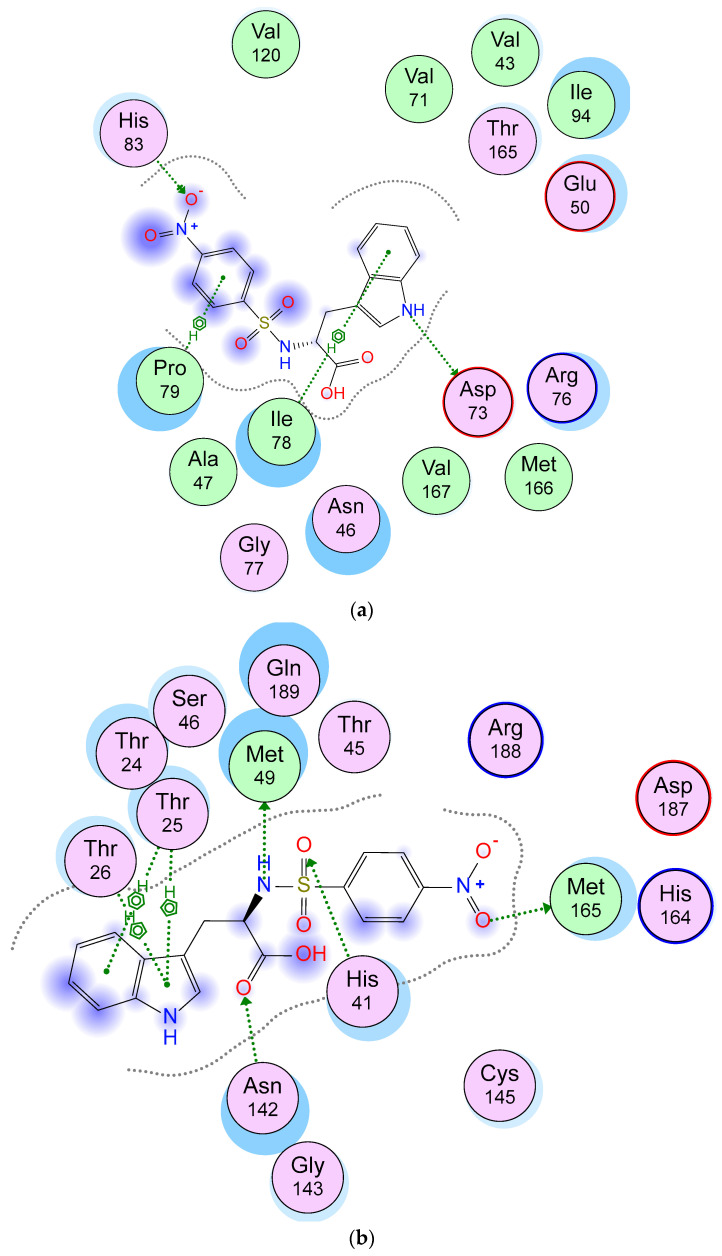
(**a**) 2D representation of the binding interactions between DNSPA and 5MMN. (**b**) 2D representation of the binding interactions between DNSPA and 6LU6.

Molecular docking studies were carried out to gain more insight into the binding mode and possible mechanism of actions of DNSPA against the COVID-19 main protease (6LU7) and DNA gyrase of E. coli (5MMN). Detailed chemical interactions of DNSPA with 5MMN and 6LU7 are shown in Table 3/Figure 6a and Table 4/Figure 6b, respectively. Applying the London dG scoring function, the binding free energies of DNSPA and the standard drug (ciprofloxacin) were found to be −6.37 and −5.38 kcal/mol, respectively. Additionally, the binding free energies of standard COVID-19 drug (remdesivir) and DNSPA are −7.74 and −6.35 kcal/mol, respectively. This indicates that the binding affinity of the synthesized compound for 5MMN is greater than that of the standard antibiotics. DNSPA was well fitted into the binding site of 5MMN (Figure 6a). As a result, the atoms of DNSPA could interact with the amino acid residues of the drug target through various chemical interactions (Table 3). Notable among these interactions are those involving ASP 73, ILE 78, and PRO 79. It has been discovered that these amino acids significantly impact the inhibitory effect of isoquinoline ethyl ureas against 5MMN [61]. The results also show that DNSPA fits snugly into the substrate-binding pocket. This is in agreement with other works reported in the literature. A study showed that a sulfonamide with indole moiety with a docking score of −8.70 kcal/mol could bind to the active site of SARS CoV 3CL protease, thereby inhibiting the activity of the enzyme. The common binding feature DSPA and SAR CoV-2 protein was the interactions between the pi-electrons of the indole moiety and Thr 25 and Thr 26. Hydrogen bond interactions were also observed between the sulfonamide oxygen and His 41, oxygen of the nitro group and Met 165, and N-atom and Met 49.

The docking scores of some carbonyl and the sulfonamide analogues with indole moiety were studied and compared (Table 5). It was observed that the carbonyl analogues had a slightly higher binding affinity than their sulfonamide counterpart, though this increase is not significant.

## 3. Materials and Methods

### 3.1. Materials and Techniques

Na_2_CO_3_ and HCl were obtained from Fluka, while 4-nitrobenzenesulfonylchloride and L- tryptophan were obtained from Sigma-Aldrich. Infrared spectra were recorded on a Bruker FT-IR spectrophotometer. The NMR data were recorded in methanol-d4 on a Bruker DPX-300 spectrometer with ^1^H at 400 MHz and ^13^C at 400 MHz. Chemical shifts, δ, are given in ppm and referenced to tetramethylsilane (TMS). The mass spectrum was collected on a Bruker MicrOTOF spectrometer in the positive ion mode using pneumatically assisted electrospray ionization: capillary voltage, 2900 V; sample cone voltage, 15 V; extraction voltage, 1 V; source temperature, 80 °C; desolvation temperature, 160 °C; cone gas flow, 100 Lh^−1^; desolvation gas flow, 100 L h^−1^; collision voltage, 2 V; and MCP voltage, 2400 V.

### 3.2. Synthesis of ((4-nitrophenyl)sulfonyl)tryptophan (DNSPA)

**Scheme 1:**
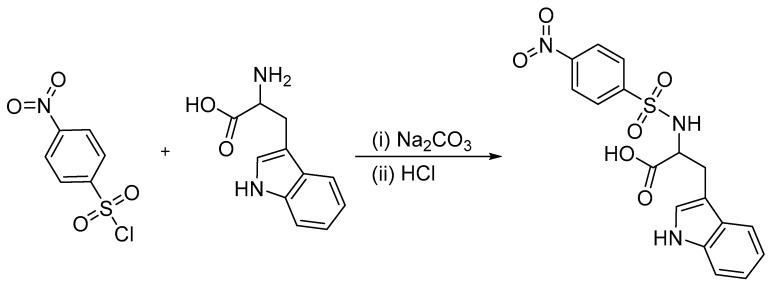
Synthesis of ((4-nitrophenyl)sulfonyl)tryptophan (DNSPA).

Na_2_CO_3_ (0.55 g, 5 mmol) was dissolved in 15 mL of water, and the solution was added to L-tryptophan (1.02 g, 5 mmol) (Scheme 1). The mixture was stirred in an ice bath until it dissolved completely. Four batches of 4-nitrobenzenesulfonylchloride (1.10 g, 5mmol) were added to the stirred mixture and maintained for one hour, after which the set-up was removed from the ice bath and the solution stirred for further 4 h. HCl (20%) was gradually added to the solution until the pH was maintained at 2. The greenish-yellow product (DNSPA) obtained was filtered, washed, and dried in the open air (Yield = 82.9%; Melting point 96.5 °C). Selected IR (υ cm^−1^): 932 (S-N str), 1125–1330 (SO_2_ str), 1458, 1478 (C-N str), 3035, 3105 (C-H str), 821, 846 (C-H bend), 777, 735 (C-S str), 3291 (N-H str), 1602 (N-H bend); ^1^H NMR (δ ppm): 2.86 (2H d), 3.22 (2H d), 3.24 (1H s), 6.94–6.85 (4H m), 7.45–7.03 (4H m), 7.77–7.74 (1H t), 8.09 (1H s); ^13^C NMR (δ ppm): 27.49, 108.23, 110.19, 116.8, 117.64, 120.21, 121.97, 122.87, 125.78, 135.66, 144.88, 148.09, 178.19. ESI-MS: [M]^+^ (*m/z)* = 388.16; UV (λmax nm) (methanol): 245 (π-π*), 290 (π-π*), 385 (n-π*), 405 (n-π*), 420 (n-π*) and 575 (n-π*). The X-ray structures and UV-Visible spectrum are presented in Figure 2, Figure 3 and Figure 5, while the FTIR, mass spec, ^1^H, and ^13^C NMR spectra of DNSPA are shown in Figures S2a and S3–S5 of the supplementary information (SI), respectively.

### 3.3. X-ray Determination of DNSPA

Green colored single crystals of DNSPA suitable for X-ray crystallography were obtained by slow evaporation of a methanolic solution of the compound, and the crystallographic data were collected on an XtaLAB Synergy, Dualflex, Pilatus 200K diffractometer at 110.2(5) K. Data reduction and absorption correction were implemented using the Crysallispro Software [62]. Using the Olex2 interface [63], the structure was solved with the ShelXT structure solution program applying intrinsic phasing and refined with the ShelXL [64] refinement package using Least Squares minimization. Anisotropic refinement was carried out on non-hydrogen atoms. In contrast, hydrogen atoms were refined using the riding model at idealized positions except for hydrogen atoms on nitrogen atoms. The positions were revealed on Fourier maps and refined isotropically. The heterocyclic fragment of molecule A shows that some of the ring’s atoms were disordered, as shown by the shape of the ADPs. There are, however, no residual electron densities around the atoms to warrant modelling of the disordered atoms. RIGU restraints were placed on the bonds around the heterocycle to minimize movement.

X-ray crystal diagrams (Figure 2a,b) are drawn with OlexDraw, and crystallographic refinement parameters are presented in Table 6. CCDC-2072038 contains the supplementary crystallographic data for this work and can be obtained through www.ccdc.cam.ac.uk/data_request/cif. (Accessed on 4 May 2022).

### 3.4. Computational Method

Computational calculations were carried out based on the DFT method with the B3LYP functional [65,66], and the 6-311++G(d,p) basis set was used for all the atoms [67]. DNSPA was optimized in the gas phase using the coordinates of the X-ray structure and also optimized in methanol using the same method based on the polarizable continuum model (PCM) [68,69]. Frequency computation of the optimized geometry was performed to ensure the structure was a true local minimum in both gas phase and solvents. The time-dependent DFT (TD-DFT) computations were also performed in methanol to simulate the electronic spectra and better understand the electronic transitions at the molecular level. Transition energies and oscillator strengths for the electronic excitation of the first 70 excited states of DNSPA were considered. The optimized structure of DNSPA in methanol was used for computing chemical shifts with the Gauge-Including Atomic Orbital method [70]. All the computations were performed at 298.15 K and 1 atm using the Gaussian 16 software [71].

### 3.5. Molecular Docking Studies

The drug targets, DNA gyrase from *E.* *coli* (PDB: 5MMN) and COVID-19 main protease (PDB: 6LU7) retrieved from the protein databank (http://www.rcsb.org, accessed on 4 May 2022) were opened in Discovery studio where the water of crystallization and unwanted side chains were removed. Molecular docking simulations of DNSPA into the binding sites of 5MMN and 6LU7 were done after the structures were energy minimized using the MMFF94x force field. The free energy of the binding interaction was calculated.

## 4. Conclusions

An indirect method capable of giving a high yield and a purer sulfonamide has been utilised in this work to synthesize a new compound, ({4-nitrophenyl}sulfonyl)tryptophan by reacting 4-nitrobenzenesulphonyl chloride and L-tryptophan. The synthesized compound’s extensive characterization was carried out and compared to the theoretical calculations for a broader understanding of the new compound’s chemistry. The X-ray crystal structure analysis of the compound revealed a four-molecule structure linked by O-H⋯O intermolecular hydrogen bonding with no significant structural differences. The Molecular packing of the crystal showed a 12-molecule supramolecular aggregate synthon stabilised by N-H⋯N and N-H⋯O intermolecular contacts. The calculated pairwise interaction energy of the dimeric structures shows a combination of electrostatic and dispersion interactions. The calculated parameters agree with experimentally observed values. Drug activity relations of the named compound were studied using in silico molecular studies, which showed reasonable chemical interactions with relevant amino acid residues of the drug targets. A slight difference exists, which is not pronounced when its activities are compared with the carbonyl derivates. Now that this compound is known, more studies in the future may include interactions with more drug targets and in vitro studies which could enable its broader applications.

## Figures and Tables

**Table 1:** Hydrogen bond geometry (Å, °).

D-H∙∙∙A	d(D∙∙∙H)	d(H∙∙∙A)	d(D∙∙∙A)	<(DHA)
N1B-H1B∙∙∙O1C	0.97(4)	1.89(4)	2.859(4)	171(4)
O3C-H3C∙∙∙O6A	0.88(6)	1.80(6)	2.678(3)	173(7)
O3D-H3D∙∙∙O6B	0.82	1.77(6)	2.590(3)	175(3)
O5A-H5A∙∙∙O4C	0.90(6)	1.71(6)	2.610(3)	173(5)
O5B-H5B∙∙∙O4D	0.82	1.86(4)	2.661(3)	169(4)

**Table 2:** Selected computed wavelength (nm), oscillator strength (f), and main orbital transition contributions (≥20%) of the absorption spectra of DNSPA.

Wavelength (nm)	Oscillator Strength (f)	Orbital Transition (%)
186	0.275	HOMO-5 → LUMO + 2 (57%) 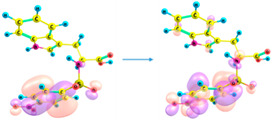
217	0.458	HOMO → LUMO + 7 (36%) 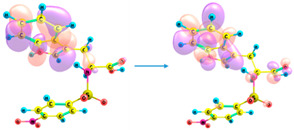
295	0.169	HOMO-4 → LUMO (50%) 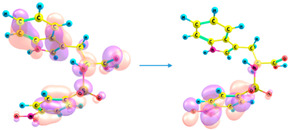
		HOMO-3 → LUMO (46%) 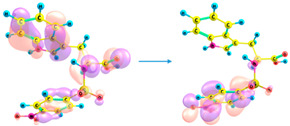

**Table 3:** Binding interactions of DNSPA with the amino acid residues of 5MMN.

Ligand	Receptor	Interaction	Distance (Å)	E (kcal/mol)
N 18	OD2 ASP 73	H-donor	2.81	−7.2
O 3	NE1 HIS 83	H-acceptor	3.69	−0.3
6-ring	CD1 ILE 78	pi-H	3.67	−0.3
6-ring	CG PRO 79	pi-H	4.38	−0.3

**Table 4:** Binding interactions of DNSPA with the amino acid residues of 6LU7.

Ligand	Receptor	Interaction	Distance (Å)	E (kcal/mol)
O 1	SD MET 165	H-donor	3.55	0.1
N 13	SD MET 49	H-donor	4.08	−2.4
O11	NE2 HIS 41	H-acceptor	3.25	−0.7
O 26	CB ASN 142	H-acceptor	3.38	−0.3
5-ring	CA THR 25	pi-H	4.22	−1.0
6-ring	CA THR 25	pi-H	4.32	−0.6
5-ring	CG2 THR 25	pi-H	4.42	−0.6
5-ring	N THR 26	pi-H	4.48	−0.9

**Table 5:** Docking scores of sulfonamide and carbonyl analogues of indole derivatives.

Scheme.	Sulfonamide Analogue	Carbonyl Analogue
1	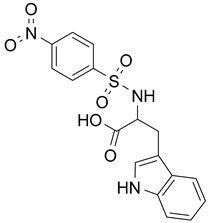 DNSPA	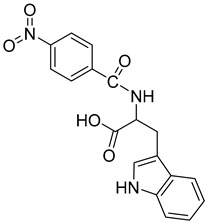 DNSPA CO- analogue
	ΔG = −6.35 kcal/mol	ΔG = −6.81 kcal/mol
2	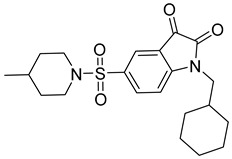 Compound 3 [6]	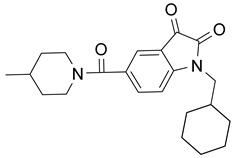
	ΔG = −7.07 kcal/mol	ΔG = −7.18 kcal/mol
3	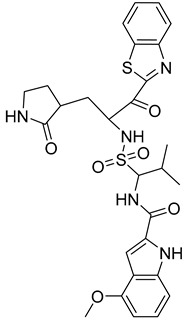 Compound 4 [6]	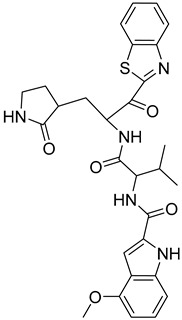
	ΔG = −7.82 kcal/mol	ΔG = −8.31 kcal/mol
4	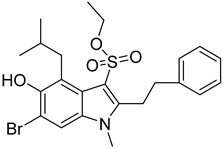 Compound 7 [6]	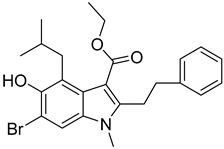
	ΔG = −7.49 kcal/mol	ΔG = −7.77 kcal/mol
5	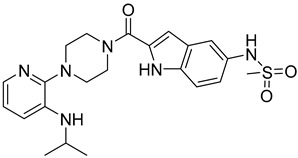 Compound 8 [6]	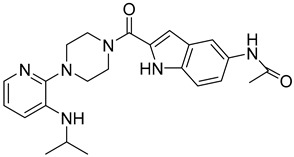
	ΔG = −7.43 kcal/mol	ΔG = −7.34 kcal/mol
6	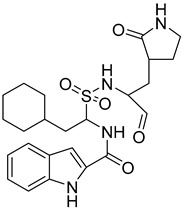 Compound 9 [6]	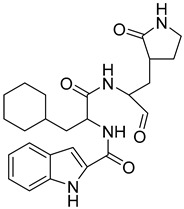
	ΔG = −8.22 kcal/mol	ΔG = −8.87 kcal/mol

**Table 6:** Crystal data and structure refinement for DNSPA.

Identification Code	DNSPA
Empirical formula	C_17_H_15_N_3_O_6_S
Formula weight	389.38
Temperature/K	110.2(5)
Crystal system	monoclinic
Space group	P2_1_
a/Å	9.61560(10)
b/Å	11.82720(10)
c/Å	29.8731(3)
α/°	90
β/°	94.9100(10)
γ/°	90
Volume/Å^3^	3384.87(6)
Z	8
ρ_calc_/g/cm^3^	1.528
μ/mm^−1^	2.092
F(000)	1616.0
Crystal size/mm^3^	0.26 × 0.26 × 0.2
Radiation	CuKα (λ = 1.54184)
2Θ range for data collection/°	5.938 to 149.56
Index ranges	−11 ≤ h ≤ 10, −14 ≤ k ≤ 13, −37 ≤ l ≤ 36
Reflections collected	27,634
Independent reflections	12167 [R_int_ = 0.0409, R_sigma_ = 0.0479]
Data/restraints/parameters	12155/28/1007
Goodness-of-fit on F^2^	1.045
Final R indexes [I >= 2σ (I)]	R_1_ = 0.0341, wR_2_ = 0.0865
Final R indexes [all data]	R_1_ = 0.0359, wR_2_ = 0.0880
Largest diff. peak/hole/e Å^−3^	0.41/−0.39
Flack parameter	−0.012(7)

## Data Availability

All data from the spectroscopic analysis and molecular docking studies are found in the manuscript and supplementary data, while CCDC-2072038 contains the supplementary crystallographic data for this work and can be obtained through www.ccdc.cam.ac.uk/data_request/cif (accessed on 4 May 2022).

## Supplementary Materials

The following supporting information can be downloaded at: https://www.mdpi.com/article/10.3390/molecules27217400/s1, Figure S1: Simulated UV spectra of DNSPA; Figure S2: (a) Experimental IR of DNSPA (b) Simulated IR of DNSPA in the gas phase; Figure S3: Mass spectra of DNSPA; Figure S4: Proton NMR of DNSPA; Figure S5: Carbon-13 NMR of DNSPA; Table S1: Experimental bond lengths for DNSPA; Table S2: Experimental bond angles for DNSPA; Table S3: Experimental torsion angles of DNSPA; Table S4: Calculated Bond lengths (Å) and bond angles (º) of optimized DNSPA in the gas phase along with its structure with atom labelling and numbering; Table S5: Vibrational assignments based on the potential energy distribution (PED) analysis of DNSPA; Table S6: Experimental and Theoretical chemical shift (ppm) of DNSPA in methanol. Refer to Table S4 for atom numbering.

## References

[1] Behmadi H., Saadati S.M., Roshani M., Ghaemy M. (2009). Synthesis of new disulphonamides from different substituted diamino pyridines. Eclética Química.

[2] Hopper D.W., Vera M.D., Howa D., Sabatini J., Xiang J.S., Ipek M., Thomason J., Hub Y., Feyfant E., Wang Q. (2009). Synthesis and biological evaluation of ((4-keto)-phenoxy)methyl biphenyl-4-sulphonamides: A class of potent aggrecanase-1 inhibitors. Bioorg. Med. Chem. Lett..

[3] Cheong M.S., Seo K.H., Chohra H., Yoon Y.E., Choe H., Kantharaj V., Lee Y.B. (2020). Influence of sulfonamide contamination derived from veterinary antibiotics on plant growth and development. Antibiotics.

[4] Nasir A.N.M., Yahaya N., Zain N.N.M., Lim V., Kamaruzaman S., Saad B., Nishiyama N., Yoshida N., Hirota Y. (2019). Thiolfunctionalized magnetic carbon nanotubes for magnetic microsolid phase extraction of sulfonamide antibiotics from milks and commercial chicken meat products. Food Chem..

[5] Supuran C.T., Casini A., Scozzafava A. (2003). Protease inhibitors of the sulfonamide type: Anticancer, anti-inflammatory, and antiviral agents. Med. Res. Rev..

[6] Wright S.W., Hallstrom K.N. (2006). A convenient preparation of heteroaryl sulfonamides and sulfonyl fluorides from heteroaryl thiols. J. Org. Chem..

[7] Eze F.U., Onyeyilim E., Ugwu D.I., Ezeokonkwo M.A., Udaya O.P., Uzoewulu C.P., Eze C.C., Okonkwo V.I. (2020). Synthesis, Characterization and Anti-Inflammatory Activities of N-Aryl Substituted Benzenesulphonamide Derivatives. Can. J. Pure Appl. Sci..

[8] Caddick S., Wilden J.D., Judd D.B. (2004). Direct synthesis of sulfonamides and activated sulfonate esters from sulfonic acids. J. Am. Chem. Soc..

[9] Frost C.G., Hartley J.P., Griffin D. (2002). Catalytic arylation of sulfamoyl chlorides: A practical synthesis of sulfonamides. Synlett.

[10] Harmata M., Zheng P., Huang C., Gomes M.G., Jing W., Ranyanil K., Balan G., Calkins N.L. (2007). Expedient synthesis of sulfinamides from sulfonyl chlorides. J. Org. Chem..

[11] Sridhar R., Srinivas B., Kumar V.P., Narender M., Rao K.R. (2007). β-Cyclodextrin-catalyzed monosulfonylation of amines and amino acids in water. Adv. Synth. Catal..

[12] Rad M.N.S., Khalafi-Nezhad A., Asrari Z., Behrouz S., Amini Z., Behrouz M. (2009). One-pot synthesis of sulfonamides from primary and secondary amine derived sulfonate salts using cyanuric chloride. Synthesis.

[13] Deedar A., Sayyeda T.A., Zainab S., Muhammad M.N., Mariya R., Tayyaba A.S., Shafia I., Muhammad R.S., Abdul H., Jamshed I. (2022). Utilization of transition metal fluoride-based solid support catalysts for the synthesis of sulfonamides: Carbonic anhydrase inhibitory activity and in silico study. RSC Adv..

[14] Huang X., Wang J., Ni Z., Wang S., Pan Y. (2014). Copper-mediated S–N formation via an oxygen-activated radical process: A new synthesis method for sulphonamides. Chem. Commun..

[15] Kumar S., Parumala R., Peddinti R.K. (2016). Metal-free synthesis of sulfonamides via iodine-catalysed oxidative coupling of sulfonyl hydrazides and amines. Tetrahedron Lett..

[16] Jafarpour M., Rezaeifard A., Golshani T. (2011). A green catalyst-free method for the synthesis of sulfonamides and sulfonylazides. Phosphorus Sulfur Silicon.

[17] Vicente D.A., Galdino D., Navarro M., Menezes P.H. (2020). Electrochemical synthesis of sulfonamides in graphite powder macro electrode. Green Chem..

[18] Jiang J.Y., Wang Q.Q., Liang S., Hu L.M., Little R.D., Zeng C.C. (2016). Electrochemical oxidative amination of sodium sulfinates: Synthesis of sulfonamides mediated by nh_4_i as a redox catalyst. J. Org. Chem..

[19] Zhang C., Chen Y., Yuan G. (2016). Electrosynthesis of arylsulfonamides from amines and sodium sulfinates using h_2_o-nai as the electrolyte solution at room temperature. Chin. J. Chem..

[20] Frontana-Uribe B.A., Little R.D., Ibanez J.G., Palmad A., Vasquez-Medrano R. (2010). Organic electrosynthesis: A promising green methodology in organic chemistry. Green Chem..

[21] Gioiello A., Rosatelli E., Teofrasti M., Filipponi P., Pellicciari R. (2013). Building a sulfonamide library by eco-friendly flow synthesis. ACS Comb. Sci..

[22] Shovan M., Suniti M. (2020). Synthesis of sulfonamide and their synthetic and therapeutic applications: Recent advances. Tetrahedron.

[23] Sonntag N.O.V. (1953). The reactions of aliphatic acid chlorides. Chem. Rev..

[24] Medebielle M., Onomura O., Keirouz R., Okada E., Yano H., Terauchi T. (2002). Indium-mediated reformatsky-type reaction of β-aminovinylchlorodifluoromethyl ketones with heteroaryl. Synthesis.

[25] Wydysh E.A., Medghalchi S.M., Vadlamudi A., Townsend C.A. (2009). Design and synthesis of small molecule glycerol 3-phosphate acyltransferase inhibitors. J. Med. Chem..

[26] Mushtaq N., Khan I.U., Yar M., Afzal S., Simpson J.N. (2012). N-Bis(2-hydroxyethyl)-4-methylbenzenesulfonamide. Acta Cryst..

[27] Olayinka O.A., Oluwole B.F., Feipeng W., Johnbull O.E., Zheng S. (2012). Room temperature synthesis and antibacterial activity of new sulfonamides containing n,n-diethyl-substituted amidomoieties. Int. J. Med. Chem..

[28] Galal A., Mahmoud K. (2021). Antiproliferative Evaluation and molecular docking studies of some sulfonyl-α-l-amino acid derivatives coupled with anisamide scaffold. Egypt. J. Chem..

[29] Taheri E., Mirjafary Z., Saeidian H. (2018). Highly efficient regioselective synthesis, spectroscopic characterizations and DFT calculations of novel hydroxymethylated 1,4-disubstituted-1,2,3-triazole-based sulfonamides. J. Mol. Struct..

[30] Bonyad S.R., Mirjafary Z., Saeidian H., Rouhani M. (2019). Efficient synthesis, spectroscopic characterization and DFT based studies of novel 1-amide 4-sulfonamide-1,2,3-triazole derivatives. J. Mol. Struct..

[31] Alizadeh M., Mirjafary Z., Saeidian H. (2020). Straightforward synthesis, spectroscopic characterizations and comprehensive DFT calculations of novel 1-ester 4-sulfonamide-1,2,3-triazole scaffolds. J. Mol. Struct..

[32] Verma S.K., Verma R., Xue F., Thakur P.K., Girish Y.R., Rakesh K.P. (2020). Antibacterial activities of sulfonyl or sulfonamide containing heterocyclic derivatives and its structure-activity relationships (SAR) studies: A critical review. Bioorg. Chem..

[33] Ul Hassan A., Sumrra S.H., Imran M., Chohan Z.H. (2022). New 3d multifunctional metal chelates of sulfonamide: Spectral, vibrational, molecular modeling, DFT, medicinal and in silico studies. J. Mol. Struct..

[34] Okafor S.N., Angsantikul P., Ahmed H. (2022). Discovery of Novel HIV Protease Inhibitors Using Modern Computational Techniques. Int. J. Mol. Sci..

[35] Obasi L.N., Oruma U.S., Al-Swaidan I.A., Ramasami P., Ezeorah C.J., Ochonogor A.E. (2017). Synthesis, Characterization and Antibacterial Studies of *N*-(Benzothiazol-2-yl)-4-chlorobenzenesulphonamide and Its Neodymium(III) and Thallium(III) Complexes. Molecules.

[36] Oruma U.S., Ukoha P.O., Uzoewulu C.P., Ndefo J.C., Ugwuoke S.C., Ukwueze N.N., Eze T.E., Ekowo L.C., Eze F.U., Chinaegbomkpa U.V. (2021). Synthesis, Biological and In Silico Studies of a Tripodal Schiff Base Derived from 2,4,6-Triamino-1,3,5-triazine and Its Trinuclear Dy(III), Er(III), and Gd(III) Salen Capped Complexes. Molecules.

[37] Brogi S., Ramalho T.C., Kuca K., Medina-Franco J.L., Valko M. (2020). Editorial: In silico Methods for Drug Design and Discovery. Front. Chem..

[38] Dimasi J.A., Hansen R.W., Grabowski H.G. (2003). The price of innovation: New estimates of drug development costs. J. Health Econ..

[39] Song C.M., Lim S.J., Tong J.C. (2009). Recent advances in computer-aided drug design. Brief. Bioinform..

[40] Paul S.M., Mytelka D.S., Dunwiddie C.T., Persinger C.C., Munos B.H., Lindborg S.R., Schacht A.L. (2010). How to improve R&D productivity: The pharmaceutical industry’s grand challenge. Nat. Rev. Drug Discov..

[41] Ton A.T., Gentile F., Hsing M., Ban F., Cherkasov A. (2020). Rapid Identification of Potential Inhibitors of SARS-CoV-2 Main Protease by Deep Docking of 1.3 Billion Compounds. Mol. Inf..

[42] Liu W., Zhu H., Niu G., Shi E., Chen J., Sun B., Chen W., Zhou H., Yang C. (2014). Synthesis, modification and docking studies of 5-sulfonyl isatin derivatives as SARS-CoV 3C-like protease inhibitors. Bioorg. Med. Chem..

[43] Beg M.A., Athar F. (2020). Anti-HIV and Anti-HCV drugs are the putative inhibitors of RNA-dependent-RNA polymerase activity of NSP12 of the SARS CoV-2 (COVID-19). Pharm. Pharmacol. Int. J..

[44] Ugwu D.I., Okoro U.C., Ukoha P.O., Okafor S., Ibezim A., Kumar N.M. (2017). Synthesis, characterization, molecular docking and in vitro antimalarial properties of new carboxamides bearing sulphonamide. Eur. J. Med. Chem..

[45] Ugwu D.I., Okoro U.C., Mishra N.K. (2018). Synthesis, characterization and in vitro antitrypanosomal activities of new carboxamides bearing quinoline moiety. PLoS ONE.

[46] Ugwu D.I., Eze F.U., Ogboo B.C., Okoro V.N., Ugwu M.C., Okafor S.N., Ayogu J.I., Attah S.I. (2019). Synthesis of multi-target benzene-sulphonamide derivatives for the treatment of trypanosomiasis. Med. Chem..

[47] Ugwu D.I., Okoro U.C., Ukoha P.O., Gupta A., Okafor S.N. (2018). Novel anti-inflammatory and analgesic agents: Synthesis, molecular docking and in vivo studies. J. Enzym. Inhib. Med. Chem..

[48] Ugwu D.I., Okoro U.C., Ahmad H. (2017). New carboxamide derivatives bearing benzenesulphonamide as a selective COX-II inhibitor: Design, synthesis and structureactivity relationship. PLoS ONE.

[49] Ugwu D.I., Okoro U.C., Mishra N.K. (2018). Synthesis, characterization and anthelmintic activity evaluation of pyrimidine derivatives bearing carboxamide and sulphonamide moieties. J. Serb. Chem. Soc..

[50] Murthy P.K., Suneetha V., Armakovi S., Armakovi S.J., Suchetan P.A., Giri L., Rao R.S. (2018). Synthesis, characterization and computational study of the newly synthetised sulfonamide molecule. J. Mol. Struct..

[51] Ozochukwu I.S., Okpareke O.C., Izuogu D.C., Ibezim A., Ujam O.T., Asegbeloyin J.N. (2021). N’-(Pyridin-3-ylmethylene) benzenesulfonohydrazide: Crystal structure, DFT, Hirshfeld surface and in silico anticancer studies. Eur. J. Chem..

[52] Bats J.W., Coppens P., Koctzle T.F. (1977). The experimental charge density in sulfur-containing molecules. A study of the deformation electron density in sulfamic acid at 78 K by X-ray and neutron diffraction. Acta Crystallogr. Sect. B.

[53] Khan I.U., Mubashar-ur-Rehman H., Aziza S., Harrison W.T.A. (2012). 3-(1H-Indol-3-yl)-2-(2-nitro-benzene-sulfonamido)-propanoic acid including an unknown solvate. Acta Cryst..

[54] Hehre W.J. (2003). A Guide to Molecular Mechanisms and Quantum Chemical Calculations Wavefunction.

[55] Jamroz M.H. (2004). Vibrational energy distribution analysis VEDA 4. Warsaw.

[56] Roeges N.P.G. (1994). Guide to the Interpretation of Infrared Spectra of Organic Structure.

[57] Asegbeloyin J.N., Izuogu D.C., Oyeka E.E., Okpareke O.C., Ibezim A. (2019). Crystal structure, non-covalent interaction and molecular docking studies of 2-{[2-phenylsulfonyl)hydrazinylidene] methyl} benzoic acid and its dysprosium catalysed cyclised product: 2-(phenyl-sulfonyl) phthalazin-1 (2H)-one. J. Mol. Struct..

[58] Bellamy L.J. (1980). The Infrared Spectra of Complex Molecules.

[59] Pinlajer K., Kleinpeter E. (1994). Carbon13 Chemical Shifts in Structure and Spectrochemical Analysis.

[60] Arshad M.N., Faidallah H.M., Asiri A.M., Kosar N., Mahmood T. (2020). Structural, spectroscopic and nonlinear optical properties of sulfonamide derivatives; experimental and theoretical study. J. Mol. Struct..

[61] Panchaud P., Bruyère T., Blumstein A.-C., Bur D., Chambovey A., Ertel E.A., Gude M., Hubschwerlen C., Jacob L., Kimmerlin T. (2017). Discovery and Optimization of Isoquinoline Ethyl Ureas as Antibacterial Agents. J. Med. Chem..

[62] CrysAlisPro Software System (2021). Rigaku Oxford Diffraction.

[63] Dolomanov O.V., Bourhis L.J., Gildea R.J., Howard J.A.K., Puschmann H. (2009). OLEX2: A complete structure solution, refinement, and analysis program. J. Appl. Cryst..

[64] Sheldrick G.M. (2015). SHELXT-Integrated Space-Group and Crystal-Structure Determination. Acta Cryst.

[65] Becke A.D. (1993). Density-functional thermochemistry III. The role of exact exchange. J. Chem. Phys..

[66] Lee C., Yang W., Parr R.G. (1988). Development of the colle-salvetti correlation-energy formula into a functional of the electron density. Phys. Rev..

[67] Krishnan R., Binkley J.S., Seeger R., Pople J.A. (1980). Self-consistent molecular orbital methods. XX. A basis set for correlated wave functions. J. Chem. Phys..

[68] Tomasi J., Persico M. (1994). Molecular interactions in solution: An overview of methods based on continuous distributions of the solvent. Chem. Rev..

[69] Simkin B.Y., Sheikhet I. (1995). Quantum Chemical and Statistical Theory of Solutions—A Computational Approach.

[70] Wolinski K., Hilton J.F., Pulay P. (1990). Efficient implementation of the gauge independent atomic orbital method for NMR chemical shift calculations. J. Am. Chem. Soc..

[71] Frisch M.J., Trucks G.W., Schlegel H.B., Scuseria G.E., Robb M.A., Cheeseman J.R., Scalmani G., Barone V., Petersson G.A., Nakatsuji H. (2016). Gaussian 16, Revision B.01.

