# Supplementary material for: Structure and Computational Studies of New Sulfonamide Compound: {(4-nitrophenyl)sulfonyl}tryptophan
Source: Molecules. 2022 Oct 31;27(21):7400. doi: 10.3390/molecules27217400 (PMC9654880; doi:10.3390/molecules27217400)
Supplement: Supplementary file 1 [file molecules-27-07400-s001.zip › molecules-1901499-supplementary.pdf]

Structure and computational studies of new sulfonamide compound: {(4-nitrophenyl)sulfonyl}tryptophan

Florence Uchenna Eze[1], Chigozie Julius Ezeorah[1,2*], Blessing Chinweotito Ogboo[1,3], Obinna Chibueze Okpareke[1,4], Lydia Rhyman [5,6], Ponnadurai Ramasami [5,6], Sunday Nwankwo Okafor[7], Groutso Tania[8], Simeon Atiga[4], Thomas Ugochukwu Ejiyi[1], Mirabel Chinasa Ugwu[1], Chiamaka Peace Uzoewulu[1,9], Jude Ikechukwu Ayogu[1], Ogechi Chinelo Ekoh[1,10], David Izuchukwu Ugwu[1*]

1 Department of Pure and Industrial Chemistry, University of Nigeria, Nsukka 410001, Nigeria
2 Department of Chemistry and Biochemistry, University of South Carolina, Columbia, SC 29208, USA
3 Department of Chemistry, State University of New York at Buffalo, Buffalo, NY 14260, USA
4 School of Science, University of Waikato, Private Bag 3105, Hamilton 3240, New Zealand
5 Computational Chemistry Group, Department of Chemistry, Faculty of Science, University of Mauritius, Réduit 808037, Mauritius
6 Centre for Natural Product Research, Department of Chemical Sciences, University of Johannesburg, Doornfontein, Johannesburg 2028, South Africa
7 Department of Pharmaceutical and Medicinal Chemistry, University of Nigeria, Nsukka 410011, Nigeria
8 School of Chemical Sciences, University of Auckland, Private Bag 92019, Auckland 1142, New Zealand
9 Department of Chemistry, North Carolina State University, Raleigh, NC 27607, USA
10 Department of Chemistry, Evangel University, Akaeze, Ebonyi State, EB 491104, Nigeria
* Correspondence: chigozie.ezeorah@unn.edu.ng (C.J.E.); izuchukwu.ugwu@unn.edu.ng (D.I.U.); Tel.: +1-803-955-6609 (C.J.E.); +234-706-087-0918 (D.I.U.)

Table S1. Experimental bond lengths for DNSPA

Atom	Atom	Length/Å	Atom	Atom	Length/Å
S1C	O2C	1.430(2)	C14B	C15B	1.381(5)
S1C	O1C	1.430(3)	C14B	C13B	1.383(5)
S1C	N1C	1.600(3)	C1C	C2C	1.526(5)
S1C	C12C	1.784(4)	C3B	C4B	1.431(5)
S1A	O2A	1.426(3)	C3B	C10B	1.363(5)
S1A	O1A	1.433(3)	C3B	C2B	1.496(5)
S1A	N1A	1.624(3)	C11D	C1D	1.522(4)
S1A	C12A	1.775(3)	C1A	C11A	1.529(5)
S1B	O1B	1.439(3)	C1A	C2A	1.531(5)
S1B	O2B	1.435(3)	C17B	C12B	1.385(5)
S1B	N1B	1.612(3)	C17B	C16B	1.396(5)
S1B	C12B	1.778(3)	C15A	C14A	1.379(5)
S1D	O2D	1.434(3)	C15A	C16A	1.379(5)
S1D	O1D	1.437(3)	C12B	C13B	1.394(5)
S1D	N1D	1.604(3)	C10D	C3D	1.363(6)
S1D	C01K	1.769(3)	C9C	C4C	1.423(5)
O5A	C11A	1.300(4)	C9C	C8C	1.391(5)
O3C	C11C	1.295(4)	C4C	C5C	1.390(5)
O4C	C11C	1.229(4)	C15B	C16B	1.377(5)
O4D	C11D	1.223(4)	C4B	C5B	1.399(5)

O5B	C11B	1.302(4)	C4B	C9B	1.416(5)
O6A	C11A	1.232(4)	C17C	C12C	1.388(5)
O3D	C11D	1.307(4)	C17C	C16C	1.382(5)
O5D	N3D	1.232(4)	C12C	C13C	1.384(5)
O6B	C11B	1.225(4)	C15C	C16C	1.379(5)
O3A	N3A	1.239(4)	C15C	C14C	1.379(6)
O6D	N3D	1.220(4)	C12A	C13A	1.384(5)
O4B	N3B	1.229(5)	C12A	C17A	1.386(5)
N3D	C016	1.472(4)	C1D	C2D	1.535(5)
O3B	N3B	1.225(4)	C2D	C3D	1.500(5)
O6C	N3C	1.217(5)	C4D	C9D	1.405(5)
N2C	C9C	1.375(5)	C4D	C3D	1.437(5)
N2C	C10C	1.373(5)	C4D	C5D	1.393(6)
N1A	C1A	1.464(5)	C14A	C13A	1.389(5)
N1C	C1C	1.462(4)	C5B	C6B	1.374(6)
O5C	N3C	1.224(5)	C9D	C8D	1.398(5)
O4A	N3A	1.224(4)	C13C	C14C	1.394(5)
N2D	C10D	1.376(5)	C9B	C8B	1.398(5)
N2D	C9D	1.380(5)	N2A	C10A	1.356(7)
N2B	C9B	1.371(5)	N2A	C9A	1.354(8)
N2B	C10B	1.380(4)	C8B	C7B	1.388(6)
N1B	C1B	1.452(5)	C17A	C16A	1.383(5)
N3C	C15C	1.484(5)	C4A	C3A	1.436(6)
N3A	C15A	1.473(4)	C4A	C5A	1.398(7)
C11C	C1C	1.533(4)	C4A	C9A	1.436(6)
N3B	C15B	1.472(4)	C5C	C6C	1.381(6)
N1D	C1D	1.462(4)	C3A	C2A	1.494(5)
C1B	C11B	1.527(4)	C3A	C10A	1.366(6)
C1B	C2B	1.530(5)	C6B	C7B	1.398(6)
C016	C017	1.383(5)	C8D	C7D	1.380(7)
C016	C01T	1.375(5)	C7D	C6D	1.399(7)
C017	C018	1.380(5)	C6D	C5D	1.393(6)
C018	C01K	1.390(5)	C8C	C7C	1.384(6)
C3C	C4C	1.433(5)	C7C	C6C	1.401(6)
C3C	C2C	1.494(4)	C5A	C6A	1.395(7)
C3C	C10C	1.362(5)	C9A	C8A	1.380(8)
C01A	C01K	1.382(5)	C6A	C7A	1.465(11)
C01A	C01T	1.388(5)	C8A	C7A	1.323(10)

Table S2. Experimental bond angles for DNSPA

Atom	Atom	Atom	Angle/°	Atom	Atom	Atom	Angle/°
O2C	S1C	N1C	105.42(15)	C8C	C9C	C4C	122.5(4)
O2C	S1C	C12C	106.60(15)	C9C	C4C	C3C	106.5(3)
O1C	S1C	O2C	120.48(16)	C5C	C4C	C3C	134.5(3)
O1C	S1C	N1C	107.43(15)	C5C	C4C	C9C	119.0(3)
O1C	S1C	C12C	106.93(16)	C14B	C15B	N3B	118.2(3)
N1C	S1C	C12C	109.75(16)	C16B	C15B	N3B	118.6(3)

O2A	S1A	O1A	120.47(16)	C16B	C15B	C14B	123.2(3)
O2A	S1A	N1A	105.23(15)	C14B	C13B	C12B	119.3(3)
O2A	S1A	C12A	107.06(16)	C5B	C4B	C3B	133.7(3)
O1A	S1A	N1A	106.85(15)	C5B	C4B	C9B	119.2(3)
O1A	S1A	C12A	107.24(15)	C9B	C4B	C3B	107.0(3)
N1A	S1A	C12A	109.74(15)	C16C	C17C	C12C	119.5(3)
O1B	S1B	N1B	108.75(16)	C17C	C12C	S1C	117.7(3)
O1B	S1B	C12B	107.27(16)	C13C	C12C	S1C	120.6(3)
O2B	S1B	O1B	120.36(16)	C13C	C12C	C17C	121.7(3)
O2B	S1B	N1B	106.15(15)	C16C	C15C	N3C	117.8(3)
O2B	S1B	C12B	106.26(16)	C14C	C15C	N3C	118.9(3)
N1B	S1B	C12B	107.43(15)	C14C	C15C	C16C	123.3(3)
O2D	S1D	O1D	120.36(16)	C016	C01T	C01A	118.6(3)
O2D	S1D	N1D	106.18(16)	C13A	C12A	S1A	118.6(3)
O2D	S1D	C01K	106.76(15)	C13A	C12A	C17A	122.1(3)
O1D	S1D	N1D	109.00(15)	C17A	C12A	S1A	119.2(3)
O1D	S1D	C01K	107.46(16)	N1D	C1D	C11D	109.6(3)
N1D	S1D	C01K	106.30(15)	N1D	C1D	C2D	112.6(3)
O5D	N3D	C016	118.0(3)	C11D	C1D	C2D	112.3(3)
O6D	N3D	O5D	123.9(3)	O5B	C11B	C1B	114.4(3)
O6D	N3D	C016	118.2(3)	O6B	C11B	O5B	124.7(3)
C10C	N2C	C9C	109.2(3)	O6B	C11B	C1B	121.0(3)
C1A	N1A	S1A	123.7(2)	C3D	C2D	C1D	111.8(3)
C1C	N1C	S1C	124.0(2)	C9D	C4D	C3D	106.9(3)
C10D	N2D	C9D	108.7(3)	C5D	C4D	C9D	118.6(3)
C9B	N2B	C10B	108.8(3)	C5D	C4D	C3D	134.5(4)
C1B	N1B	S1B	125.4(2)	C15A	C14A	C13A	118.1(3)
O6C	N3C	O5C	124.5(4)	C6B	C5B	C4B	118.3(4)
O6C	N3C	C15C	118.0(3)	C3C	C2C	C1C	113.1(3)
O5C	N3C	C15C	117.5(4)	N2D	C9D	C4D	107.7(3)
O3A	N3A	C15A	118.1(3)	N2D	C9D	C8D	129.2(4)
O4A	N3A	O3A	123.7(3)	C8D	C9D	C4D	123.2(4)
O4A	N3A	C15A	118.2(3)	C12C	C13C	C14C	119.0(4)
O3C	C11C	C1C	116.5(3)	C3C	C10C	N2C	109.9(3)
O4C	C11C	O3C	124.5(3)	N2B	C9B	C4B	107.4(3)
O4C	C11C	C1C	119.0(3)	N2B	C9B	C8B	130.1(3)
O4B	N3B	C15B	118.0(3)	C8B	C9B	C4B	122.5(3)
O3B	N3B	O4B	124.0(3)	C10D	C3D	C2D	126.9(3)
O3B	N3B	C15B	118.1(3)	C10D	C3D	C4D	106.8(3)
C1D	N1D	S1D	123.7(2)	C4D	C3D	C2D	126.3(3)
N1B	C1B	C11B	109.1(3)	O5A	C11A	C1A	114.0(3)
N1B	C1B	C2B	113.3(3)	O6A	C11A	O5A	124.1(3)
C11B	C1B	C2B	111.0(3)	O6A	C11A	C1A	122.0(3)
C017	C016	N3D	117.2(3)	C9A	N2A	C10A	108.9(4)
C01T	C016	N3D	119.4(3)	C15C	C16C	C17C	118.2(4)
C01T	C016	C017	123.4(3)	C12A	C13A	C14A	119.0(3)
C018	C017	C016	117.8(3)	C7B	C8B	C9B	116.5(3)
C017	C018	C01K	119.7(3)	C3B	C10B	N2B	110.0(3)

C4C	C3C	C2C	126.0(3)	C16A	C17A	C12A	119.1(3)
C10C	C3C	C4C	107.2(3)	C3B	C2B	C1B	112.7(3)
C10C	C3C	C2C	126.8(3)	C5A	C4A	C3A	133.9(4)
C01K	C01A	C01T	118.9(3)	C5A	C4A	C9A	121.9(4)
C15B	C14B	C13B	118.4(3)	C9A	C4A	C3A	104.2(4)
N1C	C1C	C11C	110.8(3)	C15A	C16A	C17A	118.2(3)
N1C	C1C	C2C	111.3(3)	C6C	C5C	C4C	118.9(4)
C2C	C1C	C11C	109.5(3)	C4A	C3A	C2A	126.3(4)
C4B	C3B	C2B	125.6(3)	C10A	C3A	C4A	107.6(4)
C10B	C3B	C4B	106.7(3)	C10A	C3A	C2A	126.1(4)
C10B	C3B	C2B	127.7(3)	C5B	C6B	C7B	122.0(4)
O4D	C11D	O3D	124.5(3)	C3A	C2A	C1A	112.2(3)
O4D	C11D	C1D	122.0(3)	C15C	C14C	C13C	118.2(3)
O3D	C11D	C1D	113.5(3)	C7D	C8D	C9D	116.5(4)
N1A	C1A	C11A	107.9(3)	C15B	C16B	C17B	118.3(3)
N1A	C1A	C2A	112.3(3)	C8D	C7D	C6D	122.0(4)
C11A	C1A	C2A	112.3(3)	C8B	C7B	C6B	121.4(4)
C12B	C17B	C16B	119.1(3)	C5D	C6D	C7D	120.5(4)
C14A	C15A	N3A	118.5(3)	C7C	C8C	C9C	116.6(4)
C14A	C15A	C16A	123.4(3)	C6D	C5D	C4D	119.2(4)
C16A	C15A	N3A	118.1(3)	C8C	C7C	C6C	122.0(4)
C17B	C12B	S1B	120.4(3)	N2A	C10A	C3A	110.2(5)
C17B	C12B	C13B	121.6(3)	C5C	C6C	C7C	121.0(4)
C13B	C12B	S1B	118.0(3)	C6A	C5A	C4A	116.8(5)
C3D	C10D	N2D	109.9(3)	N2A	C9A	C4A	109.0(4)
C018	C01K	S1D	116.6(3)	N2A	C9A	C8A	130.7(6)
C01A	C01K	S1D	121.7(3)	C8A	C9A	C4A	120.3(6)
C01A	C01K	C018	121.7(3)	C5A	C6A	C7A	119.1(6)
N2C	C9C	C4C	107.3(3)	C7A	C8A	C9A	118.6(6)
N2C	C9C	C8C	130.3(3)	C8A	C7A	C6A	123.2(5)

Table S3. Experimental torsion angles of DNSPA

Atom	Atom	Atom	Atom	**Angle/°**
O2C	S1C	N1C	H1C	-61.7
O2C	S1C	N1C	C1C	167.2(3)
O1C	S1C	N1C	H1C	168.7
O1C	S1C	N1C	C1C	37.5(3)
C12C	S1C	N1C	H1C	52.8
C12C	S1C	N1C	C1C	-78.4(3)
O2C	S1C	C12C	C17C	38.9(3)
O2C	S1C	C12C	C13C	-138.6(3)
O1C	S1C	C12C	C17C	169.0(3)
O1C	S1C	C12C	C13C	-8.5(3)
N1C	S1C	C12C	C17C	-74.8(3)

N1C	S1C	C12C	C13C	107.7(3)
H3C	O3C	C11C	O4C	1(5)
H3C	O3C	C11C	C1C	-178(5)
H2C	N2C	C9C	C4C	179.1
H2C	N2C	C9C	C8C	-0.6
C10C	N2C	C9C	C4C	-0.8(4)
C10C	N2C	C9C	C8C	179.4(4)
H2C	N2C	C10C	C3C	-178.1
H2C	N2C	C10C	H10C	1.9
C9C	N2C	C10C	C3C	1.8(4)
C9C	N2C	C10C	H10C	-178.1
S1C	N1C	C1C	C11C	-110.3(3)
S1C	N1C	C1C	H1CA	8.5
S1C	N1C	C1C	C2C	127.6(3)
H1C	N1C	C1C	C11C	118.7
H1C	N1C	C1C	H1CA	-122.6
H1C	N1C	C1C	C2C	-3.4
O6C	N3C	C15C	C16C	8.2(5)
O6C	N3C	C15C	C14C	-171.5(4)
O5C	N3C	C15C	C16C	-171.6(4)
O5C	N3C	C15C	C14C	8.7(5)
O3C	C11C	C1C	N1C	18.5(4)
O3C	C11C	C1C	H1CA	-100.3
O3C	C11C	C1C	C2C	141.6(3)
O4C	C11C	C1C	N1C	-161.4(3)
O4C	C11C	C1C	H1CA	79.8
O4C	C11C	C1C	C2C	-38.2(4)
C2C	C3C	C4C	C9C	-178.8(3)
C2C	C3C	C4C	C5C	1.1(6)
C10C	C3C	C4C	C9C	1.5(4)
C10C	C3C	C4C	C5C	-178.7(4)
C4C	C3C	C2C	C1C	-71.3(4)
C4C	C3C	C2C	H2CA	167.4
C4C	C3C	C2C	H2CB	50
C10C	C3C	C2C	C1C	108.4(4)
C10C	C3C	C2C	H2CA	-12.9
C10C	C3C	C2C	H2CB	-130.3
C4C	C3C	C10C	N2C	-2.0(4)
C4C	C3C	C10C	H10C	177.9
C2C	C3C	C10C	N2C	178.2(3)
C2C	C3C	C10C	H10C	-1.8
N1C	C1C	C2C	C3C	-60.3(4)
N1C	C1C	C2C	H2CA	61
N1C	C1C	C2C	H2CB	178.4
C11C	C1C	C2C	C3C	176.8(3)
C11C	C1C	C2C	H2CA	-61.8
C11C	C1C	C2C	H2CB	55.5
H1CA	C1C	C2C	C3C	58.8

H1CA	C1C	C2C	H2CA	-179.8
H1CA	C1C	C2C	H2CB	-62.5
N2C	C9C	C4C	C3C	-0.4(4)
N2C	C9C	C4C	C5C	179.7(3)
C8C	C9C	C4C	C3C	179.4(3)
C8C	C9C	C4C	C5C	-0.5(5)
N2C	C9C	C8C	H8C	0.6
N2C	C9C	C8C	C7C	-179.4(4)
C4C	C9C	C8C	H8C	-179.1
C4C	C9C	C8C	C7C	0.9(6)
C3C	C4C	C5C	H5C	-0.1
C3C	C4C	C5C	C6C	-180.0(4)
C9C	C4C	C5C	H5C	179.8
C9C	C4C	C5C	C6C	-0.1(5)
H17C	C17C	C12C	S1C	3.2
H17C	C17C	C12C	C13C	-179.3
C16C	C17C	C12C	S1C	-176.9(3)
C16C	C17C	C12C	C13C	0.6(6)
H17C	C17C	C16C	C15C	-179.3
H17C	C17C	C16C	H16C	0.7
C12C	C17C	C16C	C15C	0.8(6)
C12C	C17C	C16C	H16C	-179.3
S1C	C12C	C13C	H13C	-4.2
S1C	C12C	C13C	C14C	175.8(3)
C17C	C12C	C13C	H13C	178.4
C17C	C12C	C13C	C14C	-1.6(6)
N3C	C15C	C16C	C17C	179.1(4)
N3C	C15C	C16C	H16C	-0.9
C14C	C15C	C16C	C17C	-1.2(6)
C14C	C15C	C16C	H16C	178.9
N3C	C15C	C14C	C13C	179.9(3)
N3C	C15C	C14C	H14C	-0.1
C16C	C15C	C14C	C13C	0.2(6)
C16C	C15C	C14C	H14C	-179.9
C12C	C13C	C14C	C15C	1.2(6)
C12C	C13C	C14C	H14C	-178.7
H13C	C13C	C14C	C15C	-178.8
H13C	C13C	C14C	H14C	1.2
C4C	C5C	C6C	C7C	0.3(6)
C4C	C5C	C6C	H6C	-179.6
H5C	C5C	C6C	C7C	-179.6
H5C	C5C	C6C	H6C	0.5
C9C	C8C	C7C	H7C	179.3
C9C	C8C	C7C	C6C	-0.6(6)
H8C	C8C	C7C	H7C	-0.7
H8C	C8C	C7C	C6C	179.3
C8C	C7C	C6C	C5C	0.1(6)
C8C	C7C	C6C	H6C	180

H7C	C7C	C6C	C5C	-179.9
H7C	C7C	C6C	H6C	0
O2A	S1A	N1A	H1A	-70.5
O2A	S1A	N1A	C1A	158.8(3)
O1A	S1A	N1A	H1A	160.4
O1A	S1A	N1A	C1A	29.6(3)
C12A	S1A	N1A	H1A	44.4
C12A	S1A	N1A	C1A	-86.3(3)
O2A	S1A	C12A	C13A	33.4(3)
O2A	S1A	C12A	C17A	-144.3(3)
O1A	S1A	C12A	C13A	164.0(3)
O1A	S1A	C12A	C17A	-13.7(3)
N1A	S1A	C12A	C13A	-80.3(3)
N1A	S1A	C12A	C17A	102.0(3)
H5A	O5A	C11A	O6A	-2(4)
H5A	O5A	C11A	C1A	178(4)
S1A	N1A	C1A	H1AA	8.2
S1A	N1A	C1A	C11A	-108.5(3)
S1A	N1A	C1A	C2A	127.3(3)
H1A	N1A	C1A	H1AA	-122.6
H1A	N1A	C1A	C11A	120.8
H1A	N1A	C1A	C2A	-3.5
O3A	N3A	C15A	C14A	-164.3(3)
O3A	N3A	C15A	C16A	17.2(5)
O4A	N3A	C15A	C14A	16.1(5)
O4A	N3A	C15A	C16A	-162.3(3)
N1A	C1A	C11A	O5A	47.9(4)
N1A	C1A	C11A	O6A	-132.2(3)
H1AA	C1A	C11A	O5A	-68.7
H1AA	C1A	C11A	O6A	111.1
C2A	C1A	C11A	O5A	172.2(3)
C2A	C1A	C11A	O6A	-8.0(5)
N1A	C1A	C2A	C3A	-55.3(4)
N1A	C1A	C2A	H2AA	65.9
N1A	C1A	C2A	H2AB	-176.4
H1AA	C1A	C2A	C3A	63.9
H1AA	C1A	C2A	H2AA	-175
H1AA	C1A	C2A	H2AB	-57.3
C11A	C1A	C2A	C3A	-177.0(3)
C11A	C1A	C2A	H2AA	-55.9
C11A	C1A	C2A	H2AB	61.8
N3A	C15A	C14A	H14A	-0.1
N3A	C15A	C14A	C13A	179.9(3)
C16A	C15A	C14A	H14A	178.3
C16A	C15A	C14A	C13A	-1.7(5)
N3A	C15A	C16A	C17A	178.4(3)
N3A	C15A	C16A	H16A	-1.6
C14A	C15A	C16A	C17A	-0.0(5)

C14A	C15A	C16A	H16A	180
S1A	C12A	C13A	C14A	-178.5(3)
S1A	C12A	C13A	H13A	1.5
C17A	C12A	C13A	C14A	-0.9(5)
C17A	C12A	C13A	H13A	179.1
S1A	C12A	C17A	H17A	-3.2
S1A	C12A	C17A	C16A	176.8(3)
C13A	C12A	C17A	H17A	179.2
C13A	C12A	C17A	C16A	-0.8(5)
C15A	C14A	C13A	C12A	2.1(5)
C15A	C14A	C13A	H13A	-178
H14A	C14A	C13A	C12A	-177.9
H14A	C14A	C13A	H13A	2.1
H2A	N2A	C10A	C3A	-178.5
H2A	N2A	C10A	H10A	1.5
C9A	N2A	C10A	C3A	1.5(6)
C9A	N2A	C10A	H10A	-178.5
H2A	N2A	C9A	C4A	180
H2A	N2A	C9A	C8A	-1
C10A	N2A	C9A	C4A	-0.0(6)
C10A	N2A	C9A	C8A	178.9(6)
C12A	C17A	C16A	C15A	1.2(5)
C12A	C17A	C16A	H16A	-178.8
H17A	C17A	C16A	C15A	-178.7
H17A	C17A	C16A	H16A	1.3
C5A	C4A	C3A	C2A	4.2(8)
C5A	C4A	C3A	C10A	-175.7(5)
C9A	C4A	C3A	C2A	-177.9(4)
C9A	C4A	C3A	C10A	2.2(5)
C3A	C4A	C5A	H5AA	-2.3
C3A	C4A	C5A	C6A	177.6(5)
C9A	C4A	C5A	H5AA	-179.9
C9A	C4A	C5A	C6A	-0.0(7)
C3A	C4A	C9A	N2A	-1.3(5)
C3A	C4A	C9A	C8A	179.6(5)
C5A	C4A	C9A	N2A	176.9(4)
C5A	C4A	C9A	C8A	-2.1(7)
C4A	C3A	C2A	C1A	-77.2(5)
C4A	C3A	C2A	H2AA	161.6
C4A	C3A	C2A	H2AB	44
C10A	C3A	C2A	C1A	102.7(5)
C10A	C3A	C2A	H2AA	-18.5
C10A	C3A	C2A	H2AB	-136.2
C4A	C3A	C10A	N2A	-2.4(5)
C4A	C3A	C10A	H10A	177.7
C2A	C3A	C10A	N2A	177.8(4)
C2A	C3A	C10A	H10A	-2.2
C4A	C5A	C6A	H6A	-177.9

C4A	C5A	C6A	C7A	2.2(7)
H5AA	C5A	C6A	H6A	2
H5AA	C5A	C6A	C7A	-177.8
N2A	C9A	C8A	H8A	3
N2A	C9A	C8A	C7A	-177.0(6)
C4A	C9A	C8A	H8A	-178.2
C4A	C9A	C8A	C7A	1.8(9)
C5A	C6A	C7A	C8A	-2.7(9)
C5A	C6A	C7A	H7A	177.4
H6A	C6A	C7A	C8A	177.5
H6A	C6A	C7A	H7A	-2
C9A	C8A	C7A	C6A	1(1)
C9A	C8A	C7A	H7A	-179.6
H8A	C8A	C7A	C6A	-179.4
H8A	C8A	C7A	H7A	0
O1B	S1B	N1B	H1B	151
O1B	S1B	N1B	C1B	19.7(3)
O2B	S1B	N1B	H1B	-78.1
O2B	S1B	N1B	C1B	150.6(3)
C12B	S1B	N1B	H1B	35.2
C12B	S1B	N1B	C1B	-96.1(3)
O1B	S1B	C12B	C17B	-0.6(3)
O1B	S1B	C12B	C13B	177.3(3)
O2B	S1B	C12B	C17B	-130.5(3)
O2B	S1B	C12B	C13B	47.4(3)
N1B	S1B	C12B	C17B	116.2(3)
N1B	S1B	C12B	C13B	-65.9(3)
H5B	O5B	C11B	O6B	-0.7
H5B	O5B	C11B	C1B	179.4
H2B	N2B	C9B	C4B	179.6
H2B	N2B	C9B	C8B	-0.6
C10B	N2B	C9B	C4B	-0.4(4)
C10B	N2B	C9B	C8B	179.4(4)
H2B	N2B	C10B	C3B	179.6
H2B	N2B	C10B	H10B	-0.5
C9B	N2B	C10B	C3B	-0.4(4)
C9B	N2B	C10B	H10B	179.6
S1B	N1B	C1B	H1BA	1.4
S1B	N1B	C1B	C11B	-115.3(3)
S1B	N1B	C1B	C2B	120.5(3)
H1B	N1B	C1B	H1BA	-130
H1B	N1B	C1B	C11B	113.3
H1B	N1B	C1B	C2B	-10.9
O4B	N3B	C15B	C14B	10.0(5)
O4B	N3B	C15B	C16B	-170.0(3)
O3B	N3B	C15B	C14B	-169.4(3)
O3B	N3B	C15B	C16B	10.6(5)
N1B	C1B	C11B	O5B	54.4(4)

N1B	C1B	C11B	O6B	-125.4(3)
H1BA	C1B	C11B	O5B	-62.3
H1BA	C1B	C11B	O6B	117.9
C2B	C1B	C11B	O5B	180.0(3)
C2B	C1B	C11B	O6B	0.1(4)
N1B	C1B	C2B	C3B	-64.7(4)
N1B	C1B	C2B	H2BA	56.5
N1B	C1B	C2B	H2BB	174.1
H1BA	C1B	C2B	C3B	54.3
H1BA	C1B	C2B	H2BA	175.6
H1BA	C1B	C2B	H2BB	-66.8
C11B	C1B	C2B	C3B	172.1(3)
C11B	C1B	C2B	H2BA	-66.6
C11B	C1B	C2B	H2BB	50.9
H14B	C14B	C15B	N3B	-1.4
H14B	C14B	C15B	C16B	178.6
C13B	C14B	C15B	N3B	178.7(3)
C13B	C14B	C15B	C16B	-1.3(6)
H14B	C14B	C13B	C12B	-179.6
H14B	C14B	C13B	H13B	0.5
C15B	C14B	C13B	C12B	0.3(5)
C15B	C14B	C13B	H13B	-179.6
C10B	C3B	C4B	C5B	178.8(4)
C10B	C3B	C4B	C9B	-1.3(4)
C2B	C3B	C4B	C5B	-1.0(6)
C2B	C3B	C4B	C9B	178.9(3)
C4B	C3B	C10B	N2B	1.0(4)
C4B	C3B	C10B	H10B	-179
C2B	C3B	C10B	N2B	-179.2(3)
C2B	C3B	C10B	H10B	0.8
C4B	C3B	C2B	C1B	106.7(4)
C4B	C3B	C2B	H2BA	-14.6
C4B	C3B	C2B	H2BB	-132.1
C10B	C3B	C2B	C1B	-73.1(5)
C10B	C3B	C2B	H2BA	165.6
C10B	C3B	C2B	H2BB	48.1
H17B	C17B	C12B	S1B	-3.3
H17B	C17B	C12B	C13B	178.9
C16B	C17B	C12B	S1B	176.7(3)
C16B	C17B	C12B	C13B	-1.2(5)
H17B	C17B	C16B	C15B	-179.9
H17B	C17B	C16B	H16B	0.1
C12B	C17B	C16B	C15B	0.2(5)
C12B	C17B	C16B	H16B	-179.8
S1B	C12B	C13B	C14B	-176.9(3)
S1B	C12B	C13B	H13B	3
C17B	C12B	C13B	C14B	0.9(5)
C17B	C12B	C13B	H13B	-179.2

N3B	C15B	C16B	C17B	-179.0(3)
N3B	C15B	C16B	H16B	1.1
C14B	C15B	C16B	C17B	1.1(6)
C14B	C15B	C16B	H16B	-178.9
C3B	C4B	C5B	H5BA	-0.7
C3B	C4B	C5B	C6B	179.2(4)
C9B	C4B	C5B	H5BA	179.3
C9B	C4B	C5B	C6B	-0.7(5)
C3B	C4B	C9B	N2B	1.0(4)
C3B	C4B	C9B	C8B	-178.8(3)
C5B	C4B	C9B	N2B	-179.0(3)
C5B	C4B	C9B	C8B	1.2(5)
C4B	C5B	C6B	H6B	179.2
C4B	C5B	C6B	C7B	-0.7(6)
H5BA	C5B	C6B	H6B	-0.8
H5BA	C5B	C6B	C7B	179.2
N2B	C9B	C8B	H8B	0
N2B	C9B	C8B	C7B	-180.0(4)
C4B	C9B	C8B	H8B	179.8
C4B	C9B	C8B	C7B	-0.2(5)
C9B	C8B	C7B	C6B	-1.2(6)
C9B	C8B	C7B	H7B	178.8
H8B	C8B	C7B	C6B	178.8
H8B	C8B	C7B	H7B	-1.2
C5B	C6B	C7B	C8B	1.7(6)
C5B	C6B	C7B	H7B	-178.3
H6B	C6B	C7B	C8B	-178.2
H6B	C6B	C7B	H7B	1.8
O2D	S1D	N1D	H1D	-85.4
O2D	S1D	N1D	C1D	143.8(3)
O1D	S1D	N1D	H1D	143.6
O1D	S1D	N1D	C1D	12.7(3)
C01K	S1D	N1D	H1D	28.1
C01K	S1D	N1D	C1D	-102.8(3)
O2D	S1D	C01K	C018	42.2(3)
O2D	S1D	C01K	C01A	-139.0(3)
O1D	S1D	C01K	C018	172.6(3)
O1D	S1D	C01K	C01A	-8.6(3)
N1D	S1D	C01K	C018	-70.8(3)
N1D	S1D	C01K	C01A	107.9(3)
H3D	O3D	C11D	O4D	-1.3
H3D	O3D	C11D	C1D	176.5
O5D	N3D	C016	C017	-14.0(5)
O5D	N3D	C016	C01T	168.5(3)
O6D	N3D	C016	C017	165.2(3)
O6D	N3D	C016	C01T	-12.2(5)
H2D	N2D	C10D	H10D	0.3
H2D	N2D	C10D	C3D	-179.8

C9D	N2D	C10D	H10D	-179.8
C9D	N2D	C10D	C3D	0.2(4)
H2D	N2D	C9D	C4D	178.8
H2D	N2D	C9D	C8D	0.6
C10D	N2D	C9D	C4D	-1.2(4)
C10D	N2D	C9D	C8D	-179.3(4)
S1D	N1D	C1D	C11D	-105.9(3)
S1D	N1D	C1D	H1DA	10.4
S1D	N1D	C1D	C2D	128.4(3)
H1D	N1D	C1D	C11D	123.2
H1D	N1D	C1D	H1DA	-120.5
H1D	N1D	C1D	C2D	-2.5
N3D	C016	C017	H017	2.9
N3D	C016	C017	C018	-177.1(3)
C01T	C016	C017	H017	-179.8
C01T	C016	C017	C018	0.2(5)
N3D	C016	C01T	C01A	177.3(3)
N3D	C016	C01T	H01T	-2.6
C017	C016	C01T	C01A	0.1(5)
C017	C016	C01T	H01T	-179.9
C016	C017	C018	H018	179.5
C016	C017	C018	C01K	-0.6(5)
H017	C017	C018	H018	-0.4
H017	C017	C018	C01K	179.5
C017	C018	C01K	S1D	179.3(3)
C017	C018	C01K	C01A	0.6(5)
H018	C018	C01K	S1D	-0.7
H018	C018	C01K	C01A	-179.4
H01A	C01A	C01K	S1D	1.1
H01A	C01A	C01K	C018	179.7
C01T	C01A	C01K	S1D	-179.0(3)
C01T	C01A	C01K	C018	-0.3(5)
H01A	C01A	C01T	C016	179.9
H01A	C01A	C01T	H01T	-0.1
C01K	C01A	C01T	C016	-0.0(5)
C01K	C01A	C01T	H01T	179.9
O4D	C11D	C1D	N1D	-148.4(3)
O4D	C11D	C1D	H1DA	95.3
O4D	C11D	C1D	C2D	-22.5(4)
O3D	C11D	C1D	N1D	33.7(4)
O3D	C11D	C1D	H1DA	-82.7
O3D	C11D	C1D	C2D	159.6(3)
N2D	C10D	C3D	C2D	-178.9(3)
N2D	C10D	C3D	C4D	0.9(4)
H10D	C10D	C3D	C2D	1
H10D	C10D	C3D	C4D	-179.2
N1D	C1D	C2D	H2DA	65.2
N1D	C1D	C2D	H2DB	-176.9

N1D	C1D	C2D	C3D	-55.8(4)
C11D	C1D	C2D	H2DA	-59
C11D	C1D	C2D	H2DB	58.9
C11D	C1D	C2D	C3D	179.9(3)
H1DA	C1D	C2D	H2DA	-176.8
H1DA	C1D	C2D	H2DB	-58.9
H1DA	C1D	C2D	C3D	62.2
C1D	C2D	C3D	C10D	-82.5(4)
C1D	C2D	C3D	C4D	97.8(4)
H2DA	C2D	C3D	C10D	156.5
H2DA	C2D	C3D	C4D	-23.3
H2DB	C2D	C3D	C10D	38.6
H2DB	C2D	C3D	C4D	-141.2
C3D	C4D	C9D	N2D	1.7(4)
C3D	C4D	C9D	C8D	180.0(4)
C5D	C4D	C9D	N2D	-177.7(3)
C5D	C4D	C9D	C8D	0.5(6)
C9D	C4D	C3D	C10D	-1.6(4)
C9D	C4D	C3D	C2D	178.2(3)
C5D	C4D	C3D	C10D	177.7(4)
C5D	C4D	C3D	C2D	-2.5(7)
C9D	C4D	C5D	C6D	0.5(6)
C9D	C4D	C5D	H5D	-179.4
C3D	C4D	C5D	C6D	-178.8(4)
C3D	C4D	C5D	H5D	1.3
N2D	C9D	C8D	H8D	-2.8
N2D	C9D	C8D	C7D	177.3(4)
C4D	C9D	C8D	H8D	179.4
C4D	C9D	C8D	C7D	-0.6(6)
C9D	C8D	C7D	H7D	179.6
C9D	C8D	C7D	C6D	-0.4(6)
H8D	C8D	C7D	H7D	-0.3
H8D	C8D	C7D	C6D	179.7
C8D	C7D	C6D	H6D	-178.5
C8D	C7D	C6D	C5D	1.4(7)
H7D	C7D	C6D	H6D	1.5
H7D	C7D	C6D	C5D	-178.6
C7D	C6D	C5D	C4D	-1.4(7)
C7D	C6D	C5D	H5D	178.5
H6D	C6D	C5D	C4D	178.5
H6D	C6D	C5D	H5D	-1.6

Table S4. Calculated Bond lengths (Å) and bond angles (°) of optimized DNSPA in the gas phase along with its structure with atom labelling and numbering.

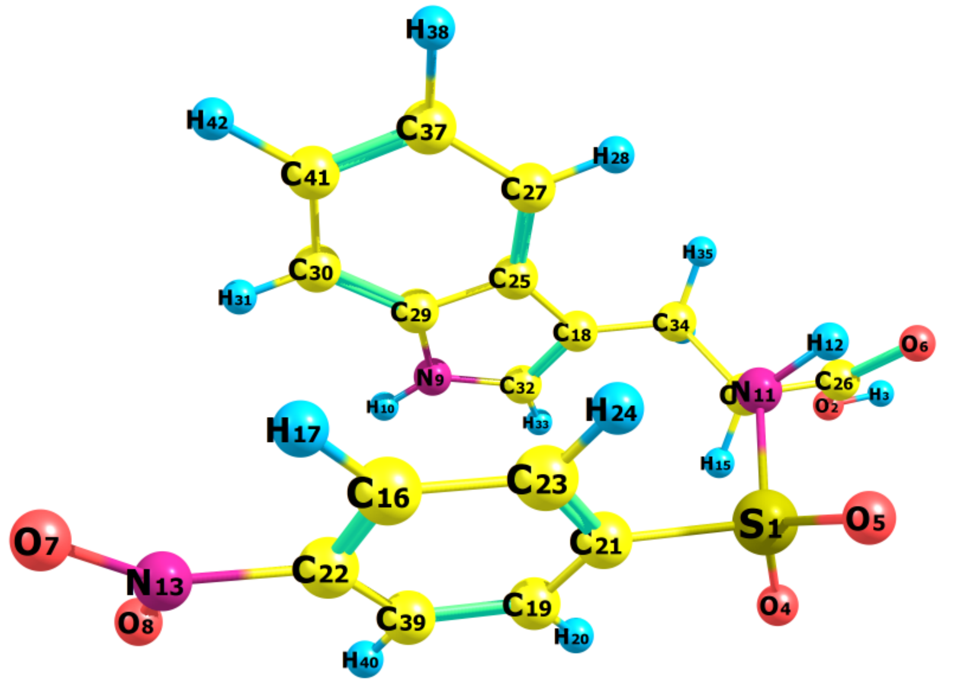

Atom labelling and numbering of the optimised structure of DNSPA in the gas phase

Bond lengths (Å)	
R(1-4)	1.462
R(1-5)	1.459
R(1-11)	1.689
R(1-21)	1.804
R(2-3)	0.970
R(2-26)	1.348
R(6-26)	1.205
R(7-13)	1.223
R(8-13)	1.225
R(9-10)	1.006
R(9-29)	1.380
R(9-32)	1.381
R(11-12)	1.019
R(11-14)	1.464
R(13-22)	1.481
R(14-15)	1.090
R(14-26)	1.527
R(14-34)	1.556
R(16-17)	1.081
R(16-22)	1.391
R(16-23)	1.389
R(18-25)	1.443
R(18-32)	1.373
R(18-34)	1.500
R(19-20)	1.082

R(19-21)	1.392
R(19-39)	1.391
R(21-23)	1.393
R(22-39)	1.390
R(23-24)	1.082
R(25-27)	1.405
R(25-29)	1.420
R(27-28)	1.084
R(27-37)	1.387
R(29-30)	1.398
R(30-31)	1.085
R(30-41)	1.388
R(32-33)	1.080
R(34-35)	1.095
R(34-36)	1.093
R(37-38)	1.084
R(37-41)	1.409
R(39-40)	1.081
R(41-42)	1.084
Bond angles (º)	
A(4-1-5)	122.3
A(4-1-11)	109.7
A(4-1-21)	106.6
A(5-1-11)	105.5
A(5-1-21)	107.7
A(11-1-21)	103.4
A(1-11-12)	107.8
A(1-11-14)	119.7
A(1-21-19)	119.1
A(1-21-23)	119.0
A(3-2-26)	107.8
A(2-26-6)	123.6
A(2-26-14)	111.8
A(6-26-14)	124.6
A(7-13-8)	124.8
A(7-13-22)	117.7
A(8-13-22)	117.5
A(10-9-29)	125.5
A(10-9-32)	125.2
A(29-9-32)	109.2
A(9-29-25)	107.2
A(9-29-30)	130.4
A(9-32-18)	110.0
A(9-32-33)	120.4
A(12-11-14)	112.6
A(11-14-15)	109.1

A(11-14-26)	109.6
A(11-14-34)	111.8
A(13-22-16)	118.7
A(13-22-39)	118.6
A(15-14-26)	107.9
A(15-14-34)	109.0
A(26-14-34)	109.4
A(14-34-18)	114.3
A(14-34-35)	108.4
A(14-34-36)	107.3
A(17-16-22)	119.9
A(17-16-23)	121.5
A(22-16-23)	118.6
A(16-22-39)	122.6
A(16-23-21)	119.2
A(16-23-24)	120.6
A(25-18-32)	106.4
A(25-18-34)	127.5
A(18-25-27)	134.1
A(18-25-29)	107.2
A(32-18-34)	126.2
A(18-32-33)	129.6
A(18-34-35)	109.9
A(18-34-36)	110.0
A(20-19-21)	120.0
A(20-19-39)	120.9
A(21-19-39)	119.1
A(19-21-23)	121.9
A(19-39-22)	118.6
A(19-39-40)	121.5
A(21-23-24)	120.3
A(22-39-40)	119.9
A(27-25-29)	118.7
A(25-27-28)	120.6
A(25-27-37)	119.1
A(25-29-30)	122.4
A(28-27-37)	120.3
A(27-37-38)	119.7
A(27-37-41)	121.2
A(29-30-31)	121.5
A(29-30-41)	117.5
A(31-30-41)	121.1
A(30-41-37)	121.1
A(30-41-42)	119.4
A(35-34-36)	106.6
A(38-37-41)	119.2

A(37-41-42)	119.5

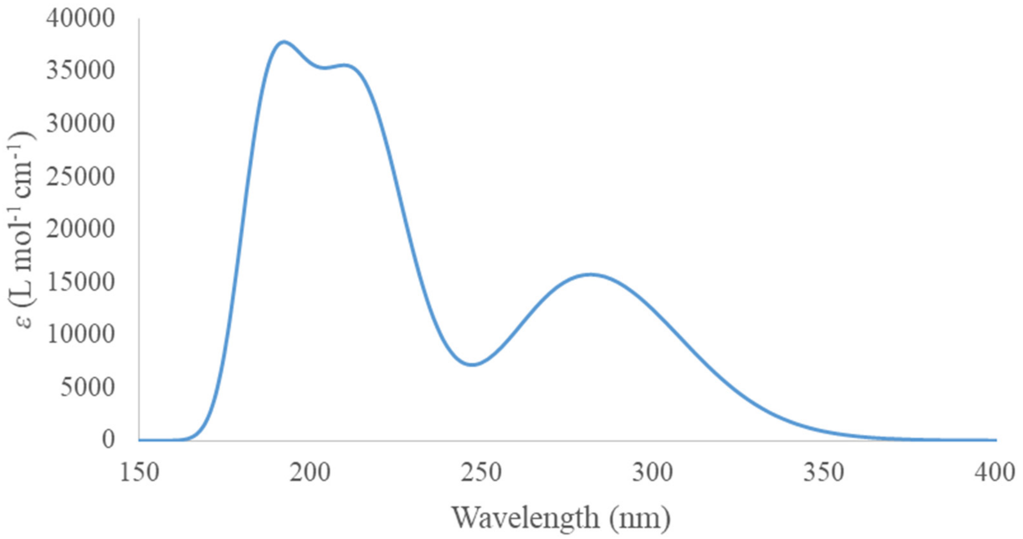

Figure S1. Simulated UV spectra of **DNSPA.**

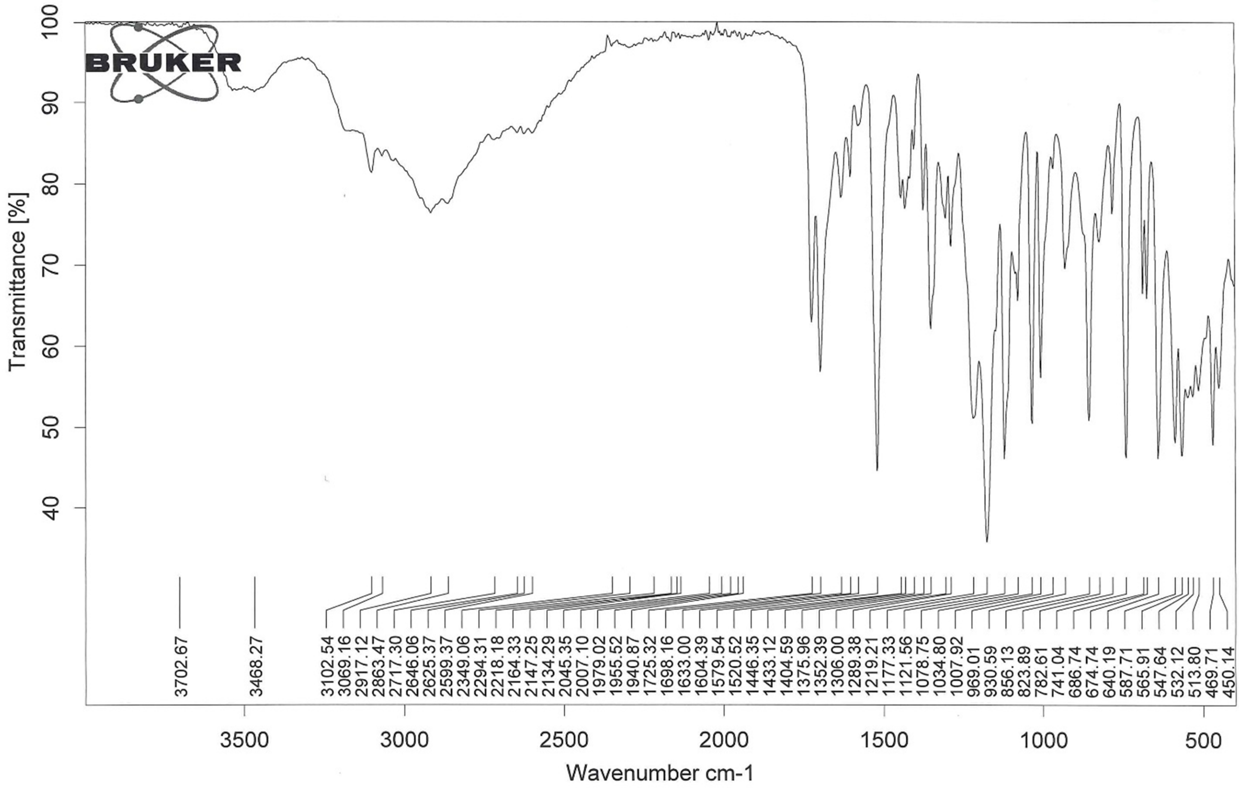

(a)

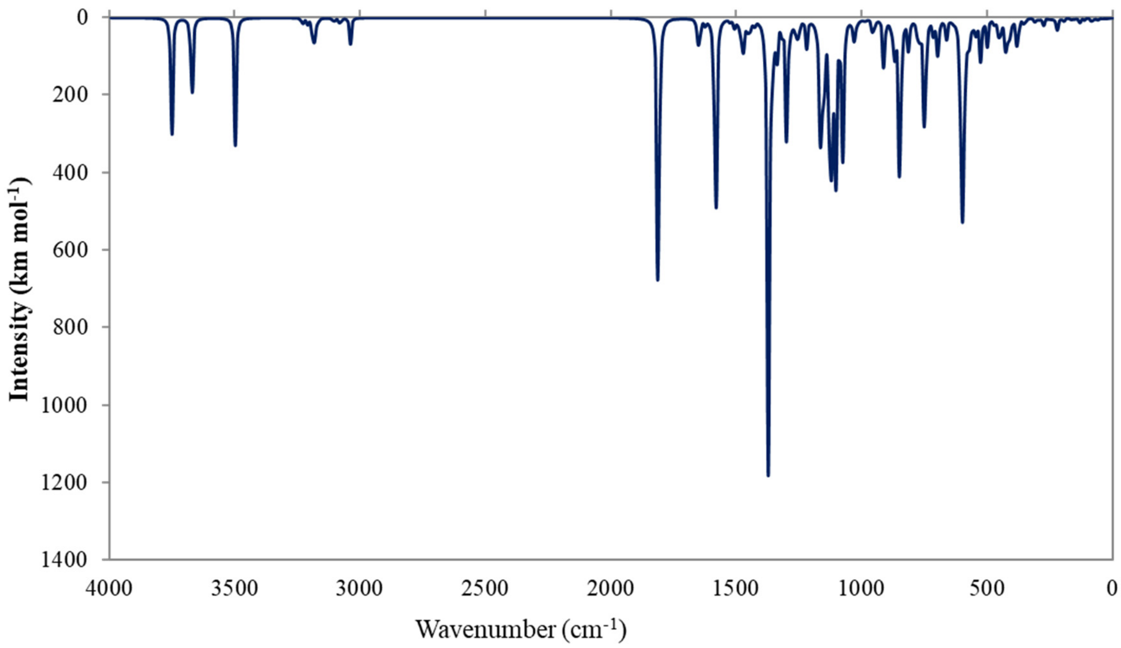

(b)

Figure S2. (a) Experimental IR of DNSPA **(b)** Simulated IR of DNSPA in the gas phase.

Table S5. Vibrational assignments based on the potential energy distribution (PED) analysis of DNSPA.

Mode	Assignments PED (≥ 10 %)	Wavenumber (cm^{-1})	Intensity (km mol^{-1})
ν_1	νOH(100)	3750	100.54
ν_2	νNH(100)	3670	78.36
ν_3	νNH(100)	3496	96.12
ν_4	νCH(99)	3236	0.22
ν_5	νCH(92)	3230	3.44
ν_6	νCH(86)	3225	2.20
ν_7	νCH(85)	3209	0.68
ν_8	νCH(83)	3208	3.61
ν_9	νCH(97)	3190	10.08
ν_{10}	νCH(97)	3180	16.64
ν_{11}	νCH(96)	3170	3.64
ν_{12}	νCH(97)	3163	0.79
ν_{13}	νCH(92)	3104	3.93
ν_{14}	νCH(89)	3078	6.22
ν_{15}	νCH(96)	3037	19.82
ν_{16}	νOC(88)	1810	282.22
ν_{17}	νCC(64)	1657	4.35
ν_{18}	νCC(76) + δCCN(11)	1648	31.51
ν_{19}	νCC(64) + δHCC(25)	1624	3.89

ν_{20}	νCC(64)	1613	0.26
ν_{21}	νCC(86)	1582	209.49
ν_{22}	νCC(45) + δHOC(12)	1580	8.51
ν_{23}	νCC(45) + δHCC(34)	1522	2.57
ν_{24}	δHCC(56) + νCC(18)	1507	6.16
ν_{25}	δHCH(82)	1492	6.30
ν_{26}	δHCC(40) + νCC(13)	1479	12.74
ν_{27}	δHNS(71)	1469	27.77
ν_{28}	δHNC(44) + νCC(18)	1449	17.89
ν_{29}	νCC(53) + δHCC(25)	1429	6.24
ν_{30}	νCC(19) + δHCC(10)	1387	4.49
ν_{31}	νCN(75) + δCCC(11)	1372	276.03
ν_{32}	νCC(55)	1370	32.54
ν_{33}	δHOC(50) + νCC(14)	1367	90.01
ν_{34}	δHCC(35) + νCC(20)	1355	7.55
ν_{35}	νCC(50) + δHCC(11)	1352	17.16
ν_{36}	δHOC(46)	1339	37.51
ν_{37}	δHOC(35) + νCC(31)	1319	0.80
ν_{38}	δHCC(65)	1317	6.44
ν_{39}	νSO(70)	1298	113.36
ν_{40}	δHNC(45)	1279	8.03
ν_{41}	δHOC(40)	1259	23.31
ν_{42}	νCC(30) + δHCC(13) + δHNC(10)	1246	5.02
ν_{43}	δHOC(53)	1219	20.88
ν_{44}	νCC(26) + δHCC(66)	1195	0.76
ν_{45}	δHCC(59)	1177	10.01
ν_{46}	νCC(32) + δHOC(14)	1161	161.90
ν_{47}	νCC(15) + δHCC(14)	1149	41.98
ν_{48}	νCC(39) + δHCC(32)	1126	66.61
ν_{49}	δHCC(16) + νCC(56)	1123	129.78
ν_{50}	δHCC(12) + νCC(57)	1116	16.41
ν_{51}	νCC(19) + δHNC(42)	1111	26.70
ν_{52}	νCC(45)	1102	113.24
ν_{53}	νCC(31)	1085	10.53
ν_{54}	νCC(72)	1074	109.88
ν_{55}	δHCC(10) + νCC(66)	1033	7.24
ν_{56}	δHCC(77)	1031	11.05
ν_{57}	νCC(15)	1018	6.49
ν_{58}	τHCCH(91)	1003	0.32
ν_{59}	τHCCN(88)	989	2.14
ν_{60}	τHCCH(87)	982	0.07
ν_{61}	νCC(40)	955	16.48
ν_{62}	τHCCC(89)	945	2.74
ν_{63}	νCC(32) + νSN(18)	911	44.11

ν_{64}	νCC(14) + δHNC(51)	888	3.32
ν_{65}	νON(11) + δCCC(53)	872	20.36
ν_{66}	τHCCC(76)	869	17.16
ν_{67}	τHCCC(86)	856	4.53
ν_{68}	νSN(23)	847	156.44
ν_{69}	τHCCN(85)	845	13.95
ν_{70}	τHNCH(79)	812	25.69
ν_{71}	νCC(15) + τHOCO(12)	780	8.55
ν_{72}	τHOCO(46)	772	8.90
ν_{73}	νCC(10) + τHOCO(35)	770	7.55
ν_{74}	τHCCC(88)	752	70.26
ν_{75}	τHCCC(72)	747	18.16
ν_{76}	δHCC(53) + νSC(21)	742	21.59
ν_{77}	τHOCO(36)	712	16.40
ν_{78}	τHCCC(84)	695	31.85
ν_{79}	δHOC(40) + τHOCC(12)	662	16.53
ν_{80}	δHCC(78)	637	0.20
ν_{81}	τHOCC(49)	634	0.83
ν_{82}	δHNC(39) + τHOCC(12)	601	184.57
ν_{83}	δHOC(16) + τHOCC(13)	597	6.26
ν_{84}	*Not assigned*	594	78.89
ν_{85}	τHNCC(80)	585	31.02
ν_{86}	τHOCC(31)	569	19.62
ν_{87}	δHCC(41)	556	5.01
ν_{88}	δCCN(49)	542	13.46
ν_{89}	δCCN(20) + τHNCH(17) + δHNC(10)	525	33.28
ν_{90}	δCNS(31)	499	20.17
ν_{91}	τHNCC(28)	470	4.02
ν_{92}	δHCC(15)	465	1.13
ν_{93}	δCCC(12) + νNC(23)	450	25.37
ν_{94}	τHNCC(89)	430	5.62
ν_{95}	τHOCC(36)	423	38.95
ν_{96}	τHCCC(91)	417	1.20
ν_{97}	δCCC(10) + δCCO(45)	407	15.30
ν_{98}	τHNCC(29)	379	31.89
ν_{99}	τHNCC(29) + δCCC(10)	351	6.38
ν_{100}	δCCO(51)	310	2.59
ν_{101}	τHNCC(33)	296	2.28
ν_{102}	τHNCH(34)	276	6.55
ν_{103}	*Not assigned*	258	0.67
ν_{104}	δCNS(57)	247	0.69
ν_{105}	δHCC(18) + τHNCH(10) + νSC(27)	233	2.25
ν_{106}	τHNCC(68)	219	9.22

ν_{107}	δHCC(15) + τHNCC(23)	196	3.22
ν_{108}	δCNS(65)	162	2.28
ν_{109}	*Not assigned*	147	0.63
ν_{110}	δCNS(60)	131	3.81
ν_{111}	δCNS(55)	114	1.88
ν_{112}	τHNCC(42)	81	4.20
ν_{113}	τHCCO(45)	64	0.64
ν_{114}	τHCCO(71)	59	0.62
ν_{115}	τHCCO(64)	55	1.22
ν_{116}	τHCCN(55)	39	0.48
ν_{117}	τHCCN(55) + τHCCS(13)	33	0.30
ν_{118}	τHCCN(55)	29	0.15
ν_{119}	τHCCN(17) + τHCCS(47)	22	0.39
ν_{120}	τHCCN(23) + τHNCH(54)	12	0.34

ν = stretching; δ = bending; δs = scissoring; ρr = rocking; w = wagging; t = twisting; τ = torsion; γ = out-of-plane.

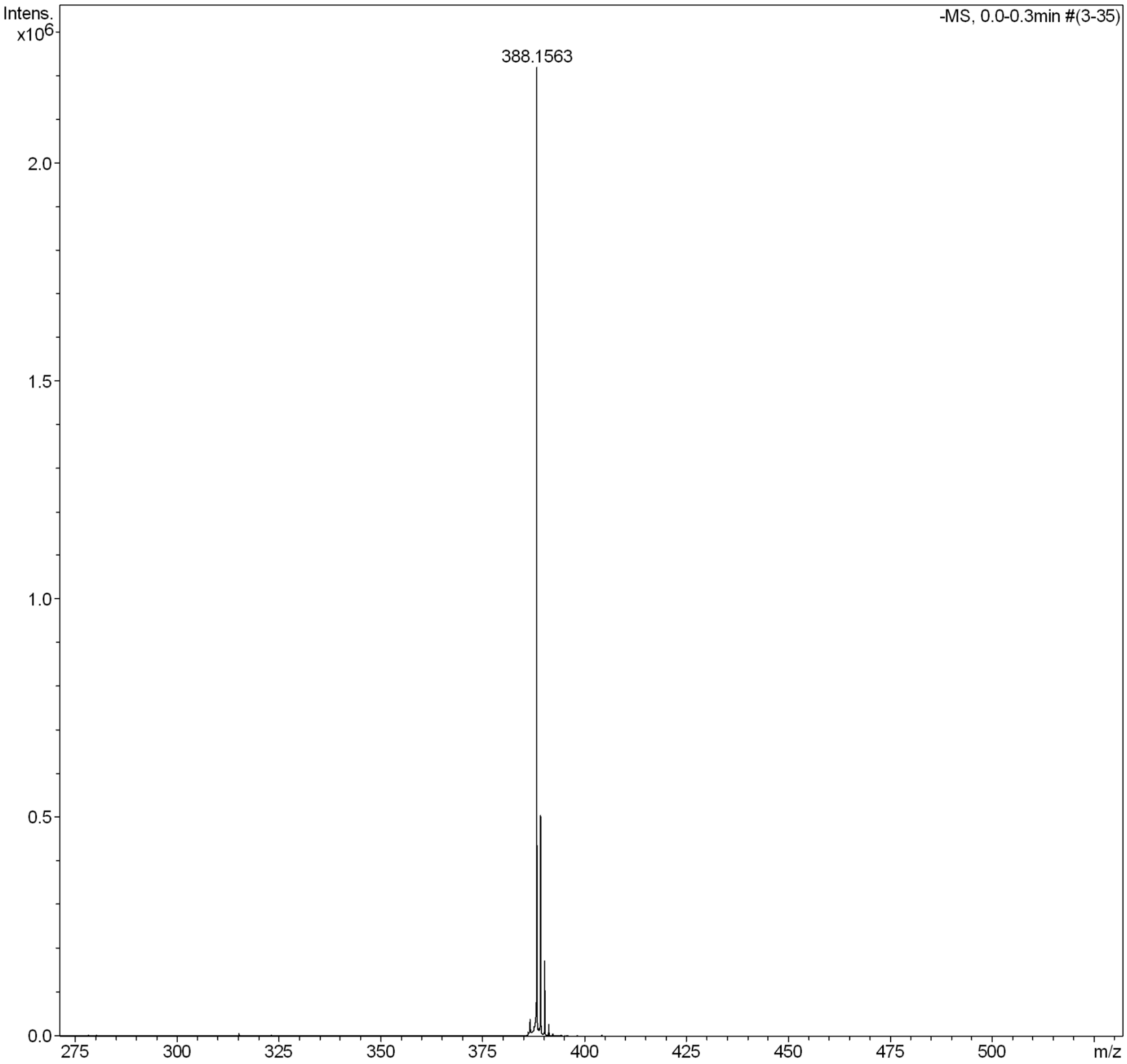

Figure S3. Mass spectra of DNSPA

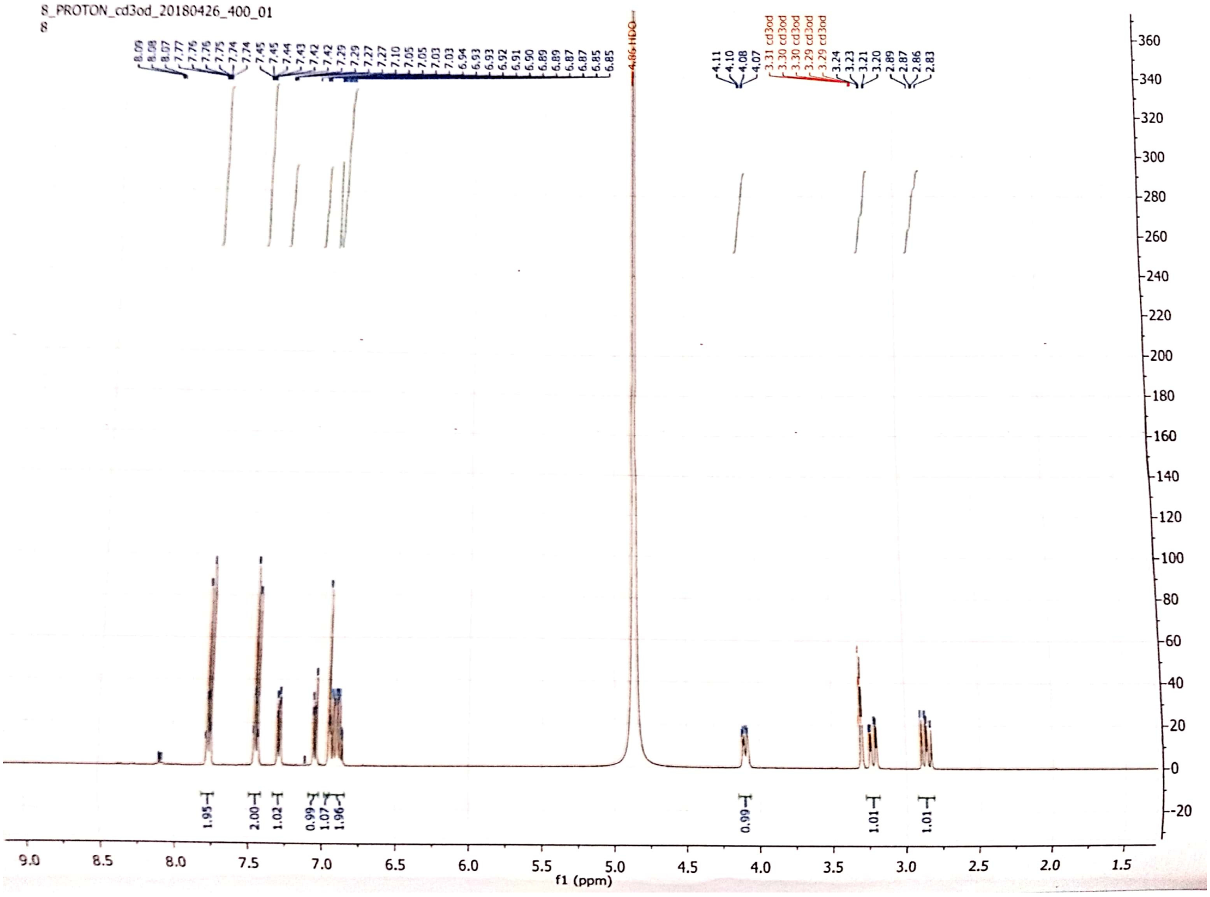

Figure S4. Proton NMR of DNSPA

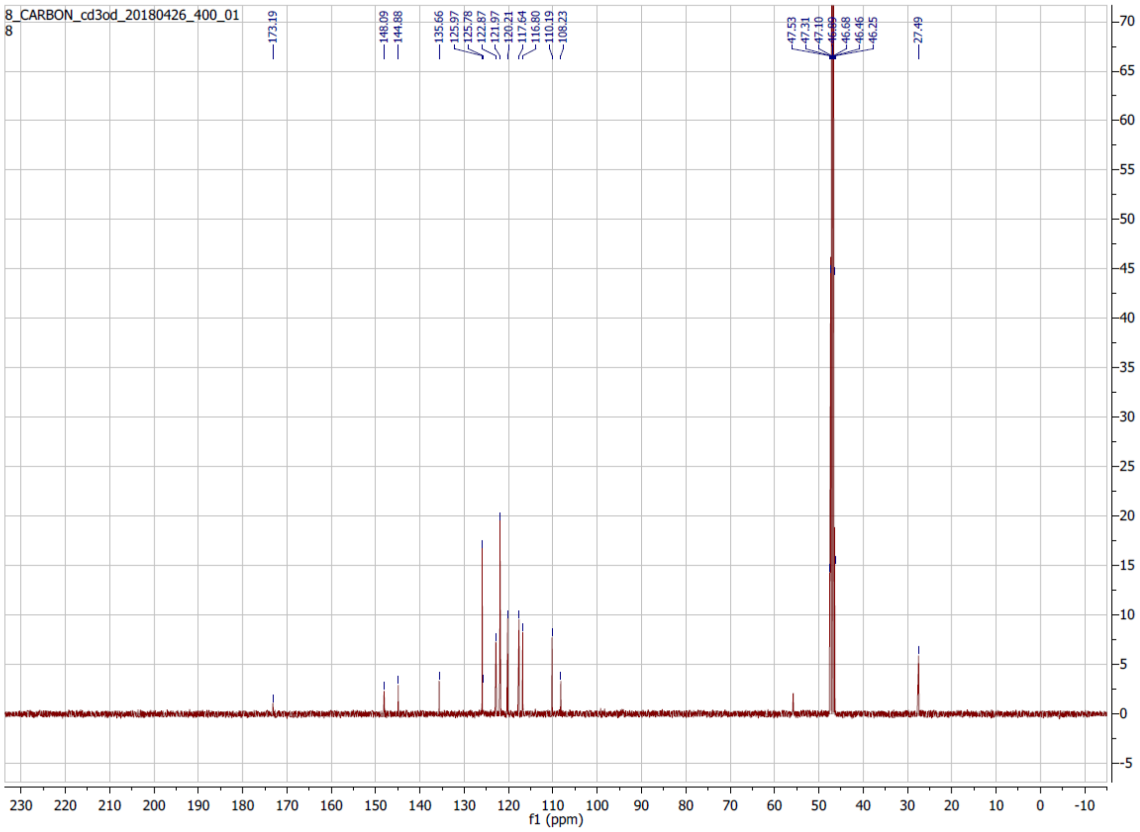

Figure S5. Carbon-13 NMR of DNSPA

Table S6. Experimental and Theoretical chemical shift (ppm) of DNSPA in methanol. Refer to Table S4 for atom numbering.

	^{1}H NMR			^{13}C NMR	
Hydrogen numbering	Experimental chemical shift (ppm)	Calculated chemical shift (ppm)	Carbon numbering	Experimental chemical shift (ppm)	Calculated chemical shift (ppm)
3	6.85	6.69	14	108.23	65.29
10	7.76	7.87	16	135.66	133.22
12	7.74	4.32	18	116.80	115.88
15	3.24	3.65	19	135.66	135.77
17	8.07	8.32	21	144.88	156.58
20	8.08	8.12	22	144.88	158.81
24	7.77	7.27	23	135.55	134.35
28	7.45	7.95	25	120.21	136.17
31	7.45	7.64	26	173.19	182.26
33	6.91	6.91	27	117.64	122.88
35	3.20	3.11	29	125.78	143.46
36	3.23	3.34	30	117.64	117.83
38	7.44	7.48	32	125.97	131.58
40	8.09	8.66	34	27.49	31.88
42	7.42	7.55	37	122.87	127.34
			39	135.66	132.19

			41	125.97	129.12
